# Multi‐proteomic profiling indicates potential regulatory signatures underlying rice resistance to *Magnaporthe oryzae*

**DOI:** 10.1111/tpj.70892

**Published:** 2026-04-21

**Authors:** Priscila A Auler, Christian Montes‐Serey, Luis W. P. Arge, Gaoyuan Song, Justin Walley, Marcio C. Silva‐Filho

**Affiliations:** ^1^ Departamento de Genética, Escola Superior de Agricultura Luiz de Queiroz Universidade de São Paulo São Paulo Brazil; ^2^ Department of Plant Pathology, Entomology, and Microbiology Iowa State University Ames Iowa USA; ^3^ Department of Agronomy and Plant Genetics University of Minnesota Minneapolis Minnesota USA

**Keywords:** *Oryza sativa*, blast disease, proteomics, phosphoproteome, acetylome

## Abstract

Omics‐driven approaches can capture systemic plant responses to pathogens. While transcriptomics is a mainstay, it alone cannot capture post‐translational modifications or protein abundance. Because of this, we employed multi‐proteomics to compare the proteome, phosphoproteome, and acetylome of two rice genotypes differing in resistance to *Magnaporthe oryzae* during early infection (12, 24, and 48 h post‐inoculation). Biochemically, the susceptible genotype displayed higher membrane lipid peroxidation. Global proteome analysis highlighted putative targets, including pathogenesis‐related 5 proteins in the resistant genotype and ASR6 (abscisic stress ripening protein family) in the susceptible genotype. Post‐translational modification landscapes diverged: the resistant genotype showed more extensive phosphorylation regulations, whereas the susceptible genotype showed more acetylation changes. Principal component analysis indicated earlier separation between mock and infected plants in the resistant genotype, consistent with faster proteome responses. Functional enrichment supported these trends: the resistant genotype was enriched for fungal defense and biotic stimulus detection, while the susceptible genotype was enriched for oxidative stress pathways. Kinase–substrate predictions linked CAMK, CK2, and SnRK2 to the largest substrate sets across genotypes and time points. Differential regulations of predicted substrates, such as Phosphatase and Tensin protein (PTEN), Synaptosome‐associated protein (SNAP), Phosphoinositide‐specific phospholipase C (PI‐PLC), and ABCG transporters, may contribute to genotype‐specific outcomes. Together, these results proposed a genotype resistance‐phosphorylation versus susceptibility‐acetylation‐associated regulatory framework, identified through discovery‐driven research, offering candidate proteins and mechanistic markers to guide functional validation and breeding strategies for improved rice blast resilience.

## Significance Statement

Despite sustained scientific efforts, rice blast remains a global challenge, and the factors distinguishing same‐species genotypes with differential resistance remain poorly understood. Using a multi‐proteomics approach integrating the global proteome and post‐translational modifications, this study identified distinct targets and biological responses between genotypes contrasting in resistance. These data suggest genotype‐specific regulatory signatures that govern resistance and provide a robust foundation for deciphering this complex plant–pathogen interaction.

## INTRODUCTION

Plant–pathogen interactions have been extensively studied over the years from various perspectives (Dodds & Rathjen, 2010; Peyraud et al., 2017). How plants recognize pathogens and their cellular responses determine their ability to overcome infection. Moreover, even within the same species, some genotypes may be resistant, while others may be susceptible. In resistant plants, basal defenses generally initiate signal transduction cascades more rapidly or stronger. This response can stop pathogen invasion and prevent further disease development (Peyraud et al., 2017). However, in susceptible plants, basal defenses are not strong enough to stop the infection, leading to disease manifestation (Künstler et al., 2016). Understanding the key differences between plants with contrasting resistance levels is essential for uncovering the mechanisms that enable their defense and recovery after damage (Mehta et al., 2008).

Different strategies have been used to identify differential plant responses in plant–pathogen interactions. Since stress responses are multidimensional and systemic, focusing initially on specific pathways may not fully capture global responses and can hinder the identification of key players and their interactions (Franco et al., 2017, 2021). In this context, omics studies provide broader insights that can later be functionally tested in a more targeted manner. Transcriptomic studies started to explore these mechanisms (Tan et al., 2009); however, transcriptional changes do not fully reflect cellular regulatory processes, as post‐transcriptional mechanisms influence the amount of active protein (Baerenfaller et al., 2008; Ghaemmaghami et al., 2003; Ponnala et al., 2014; Schwanhüusser et al., 2011; Walley et al., 2013, 2016). Therefore, complementary approaches, such as proteome‐based expression profiling to quantify protein abundance and post‐translational modifications (PTMs), are essential for obtaining a comprehensive understanding (Elmore et al., 2021).

PTMs can involve simple chemical changes, such as the addition of a phosphate, acetyl, methyl, or hydroxyl group, or more complex structural alterations. Phosphorylation and acetylation are key PTMs that regulate protein activity, stability, localization, and interaction partners, activating or deactivating signaling cascades or reshaping surface structures that interact with the plant host, acting in the plant defense (Suskiewicz, 2024). By identifying sites of phosphorylation and acetylation associated with specific stress responses, it is possible to uncover dynamic regulatory networks that underlie plant adaptation and defense. For instance, several kinases involved in phosphorylation mediate the regulation of enzymes critical to plant–fungus interactions, influencing signal transduction pathways associated with appressorium formation, cell‐wall remodeling, and stress responses (Cruz‐Mireles et al., 2024; Tan et al., 2023; Zhang et al., 2024). Protein acetylation also plays a central role in regulating gene expression. In fungi, acetyltransferases such as MoRtt109 from *Magnaporthe oryzae* can acetylate both histone and non‐histone proteins, contributing to autophagy processes related to pathogenicity (Yang et al., 2021).

In this study, we examine the complex rice‐*M. oryzae* pathosystem. Specifically, we use quantitative proteomics to compare the proteome, phosphoproteome, and acetylome of two rice genotypes that differ in resistance to *M. oryzae*. Currently, the most economical and effective approach to controlling this disease in a short time is developing rice cultivars with broad‐spectrum resistance (*R*) genes (Anon He et al., 2022). Although *R* genes provide a certain level of resistance to rice blast, they are often insufficient to prevent significant crop losses and are easily overcome due to the high genetic variability of the fungal population (Niks et al., 2015; Zhu et al., 2016). Thus, achieving more durable control of rice blast is urgently needed. Identifying proteins and PTMs associated with the rice resistance response is valuable for functional studies and for identifying breeding markers or genome‐editing targets. While detecting transient changes in protein phosphorylation and acetylation elicited by infection, comparing susceptible and resistant genotypes enables the identification of biomarkers associated with heritable differences (Fonseca de Lima et al., 2025). Our findings suggest that the resistant genotype recognizes the pathogen more rapidly and activates defense‐related proteins in a more intense and coordinated manner. At the PTM level, resistance appears to be associated with increased protein phosphorylation, whereas susceptibility is linked to elevated protein acetylation regulations. Since the pathogen is the same in both genotypes, the mechanisms underlying these distinct post‐translational responses in rice remain unclear and may reflect a proteomic signature that characterizes resistance versus susceptibility. While further investigation is required to fully elucidate the phospho‐ and acetyl‐regulatory networks, our findings reveal clear molecular differences that may serve as a valuable resource for accelerating breeding programs and guiding genome‐editing strategies to improve blast resistance in rice.

## RESULTS AND DISCUSSION

### Oxidative stress levels: Susceptible genotype experiences higher membrane damage than the resistant one during the first hours after inoculation

During pathogen–host interactions, pathogens secrete large amounts of reactive oxygen species (ROS) to breach plant barriers and facilitate penetration. If the plant does not promptly activate antioxidant and repair mechanisms, ROS‐driven oxidative damage can compromise cuticle and cell‐wall integrity, enabling more efficient fungal entry (Liu & Zhang, 2022). Guided by this framework, we assessed oxidative stress during *M. oryzae* infection by measuring hydrogen peroxide (H_2_O_2_) levels and lipid peroxidation in contrasting rice genotypes, aiming to determine whether differences in oxidative damage align with susceptibility or resistance. Thus, differences in oxidative damage can already be detected at early stages of infection and, to some extent, may allow early differentiation of resistance levels among genotypes even in the absence of visible symptoms.

**Figure 1:**
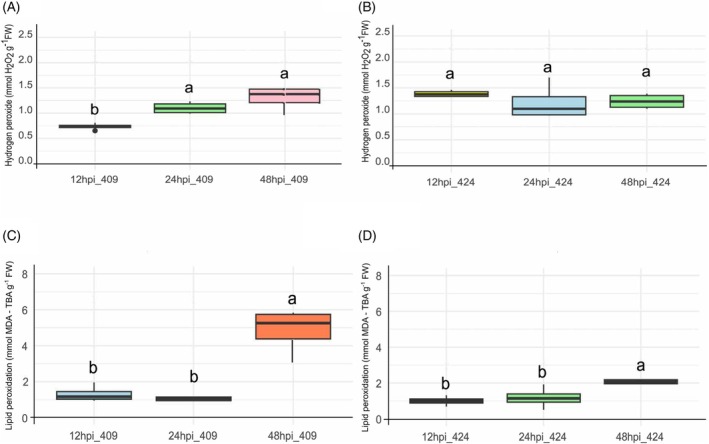
Hydrogen peroxide concentration and lipid peroxidation in rice genotypes IRGA 409 and IRGA 424 following inoculation with *Magnaporthe oryzae*. Relative concentration of hydrogen peroxide (μmol H2O2 g^−1^ FW) in leaves of IRGA 409 (A) and IRGA 424 (B) at 12, 24, and 48 h post‐inoculation (hpi). Lipid peroxidation levels (μmol MDA‐TBA g^−1^ FW) in leaves of IRGA 409 (C) and IRGA 424 (D) at 12, 24, and 48 hpi. In each boxplot, the central line represents the average, the box spans the 25th to 75th percentiles, and the whiskers indicate the minimum and maximum values (*n* = 4). Statistical significance was determined by ANOVA followed by Tukey's test (*P* < 0.05). Means labeled with the same letter within each time point are not significantly different. All treatments were compared to their respective Mock controls (mock‐inoculated plants at each time point).

The susceptible genotype, IRGA 409, exhibited a significant increase in H_2_O_2_ relative to its mock controls at 24 and 48 h post‐inoculation (Figure 1A), whereas the resistant genotype showed no statistically significant differences at any evaluated time point (Figure 1B). Both genotypes displayed increased lipid peroxidation at 48 hpi; in the resistant genotype, this increase was approximately twofold compared to 12 and 24 hpi (Figure 1D), while in the susceptible genotype, it was approximately fivefold (Figure 1C). These patterns are consistent with their known phenotypes (Anon SOSBAI‐Sociedade Sul‐Brasileira de Arroz Irrigado, 2022) and indicate that oxidative stress is more pronounced in the susceptible genotype during the early stages of infection.

Together, these biochemical data confirm that the susceptible genotype (IRGA 409) experiences higher membrane damage than IRGA 424 during the first hours after inoculation, a critical window for infection establishment. Higher lipid peroxidation in these early hours may indicate an increased ease of pathogen penetration because peroxidation, associated with oxidative stress, degrades membrane lipids and makes them more unstable and permeable (Mao, 2012). However, a direct causal link between elevated early lipid peroxidation and increased fungal penetration remains unresolved, as lipid peroxides can also act as a signaling mechanism to activate defense responses (Liu & Zhang, 2022). In agreement with the disease symptom analysis at 3 and 14 days post‐inoculation, which revealed larger and more severe lesions in the susceptible genotype (Figure S1), this may be associated with reduced antioxidant systems and repair mechanisms that may have been compromised from the onset of infection.

### Global proteomics: The resistant genotype exhibits an earlier proteomic response and accumulates PR5 proteins after infection

To investigate the temporal dynamics of the rice response to *M. oryzae*, a global quantitative proteomic analysis was performed using liquid chromatography‐tandem mass spectrometry (LC–MS/MS). Leaf samples from both genotypes (IRGA 409 and IRGA 424) were collected at 12, 24, and 48 h post‐inoculation (hpi) under both infected and mock conditions, and protein abundances were compared across treatments and time points. This approach aimed to identify patterns of differential protein accumulation and time responses associated with resistance/susceptibility.

**Figure 2:**
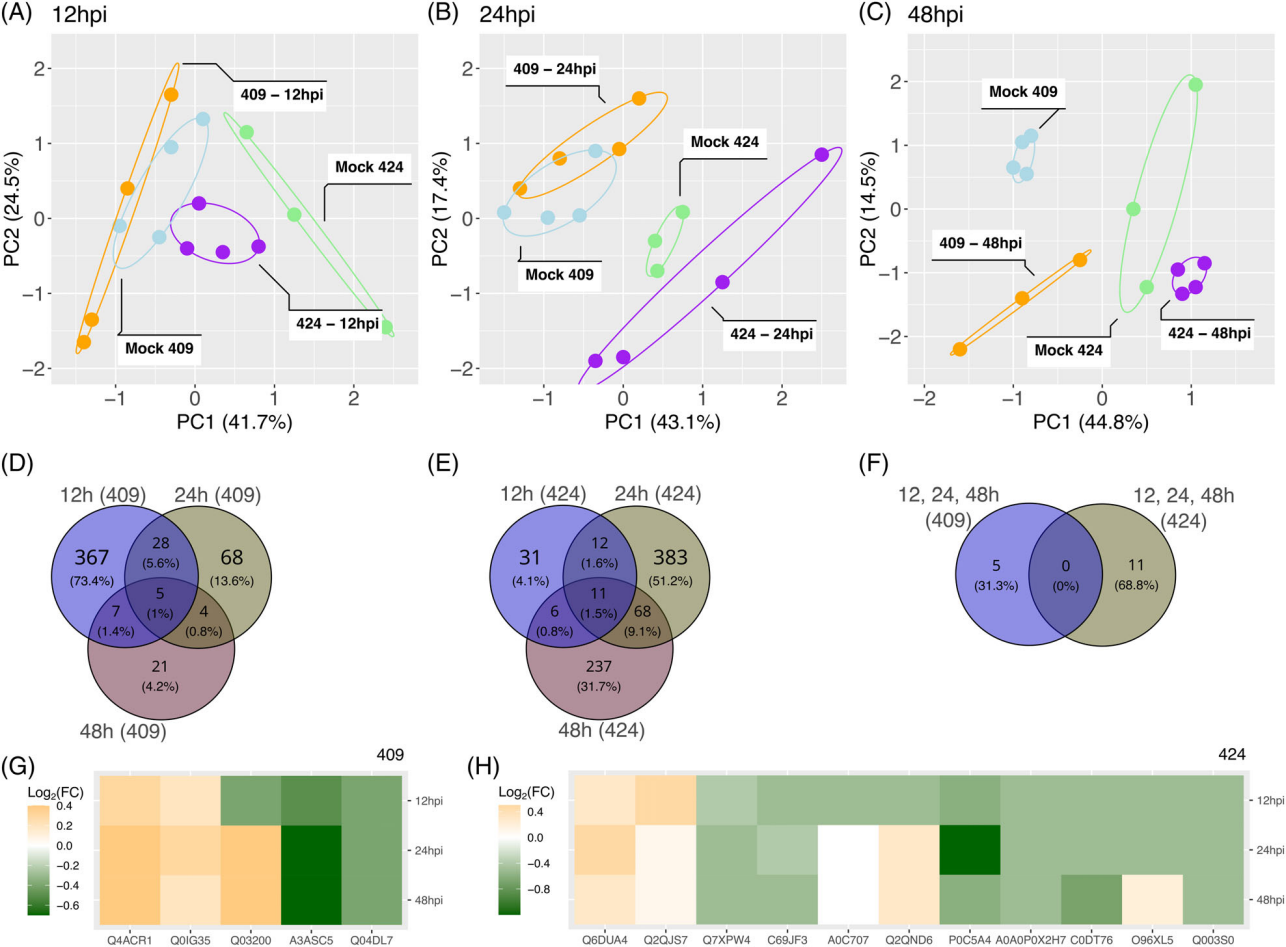
Principal component analysis (PCA) and differentially accumulated proteins (DAP) in rice genotypes IRGA 409 and IRGA 424 infected with *Magnaporthe oryzae*. PCA of IRGA 409 (blue—Mock; red—inoculated) and IRGA 424 (green—Mock; purple—inoculated) at 12 h post‐inoculation (12 hpi) (A). PCA of IRGA 409 and IRGA 424 at 24 hpi (B). PCA of IRGA 409 and IRGA 424 at 48 hpi (C). Venn diagram of DAP proteins in IRGA 409 at 12, 24, and 48 hpi (D). Venn diagram of DAP proteins in IRGA 424 at 12, 24, and 48 hpi (E). Venn diagram comparing the 5 DAPs identified at all three time points in IRGA 409 and 11 in IRGA 424 (F). The overlapping region represents proteins shared across conditions. Heatmaps showing 5 DAPs shared across all time points in IRGA 409 (G) and 11 in IRGA 424 (H). The Log2 fold change values are relative to Mock controls.

Principal component analysis (PCA) consistently distinguished the temporal and genotype‐specific nature of the proteomic response. At 12 hpi, the susceptible genotype IRGA 409 showed no clear separation between mock and infected treatments, whereas the resistant genotype IRGA 424 had already formed distinct clusters, despite the short inoculation time (Figure 2A). This pattern persisted at 24 hpi, with mock and inoculated samples remaining in separate clusters, while IRGA 409 treatments continued to overlap (Figure 2B). Only at 48 hpi were mock and infected samples from both genotypes clearly separated into distinct groups (Figure 2C). Taken together, these PCA patterns indicate that IRGA 424 exhibits an earlier and more sustained separation of proteomic states between mock and infected conditions, whereas IRGA 409 achieves a comparable separation only later at 48 hpi.

Consistent with these trends, the number of differentially accumulated proteins (DAPs) varied over time and across genotypes. In IRGA 409, 407, 105, and 37 DAPs were identified at 12, 24, and 48 hpi, respectively, whereas IRGA 424 exhibited 60, 474, and 322 DAPs at the same time points (Figure 2D,E). Five proteins were consistently differentially accumulated across all three time points (relative to their respective mocks) in IRGA 409, while 11 were in IRGA 424 (Figure 2F). Among the IRGA 409 common DAPs, protein A3ASQ5 (nuclease activity) decreased in abundance at 24 and 48 hpi, whereas Q94DL7 (abscisic acid protein family) increased at those time points (Figure 2G). In IRGA 424, notable common DAPs included Q2QND6 (pathogenesis‐related protein 5—osmotin‐like) and Q2QLS7 (pathogenesis‐related thaumatin‐like protein), both of which increased at 24 and 48 hpi, while P0C5A4 (LEA19) decreased significantly at 24 hpi (Figure 2H). The complete list of identified proteins, the DAPs in each genotype and time point, and the DAPs common across all three time points is provided in Data S1.

**Figure 3:**
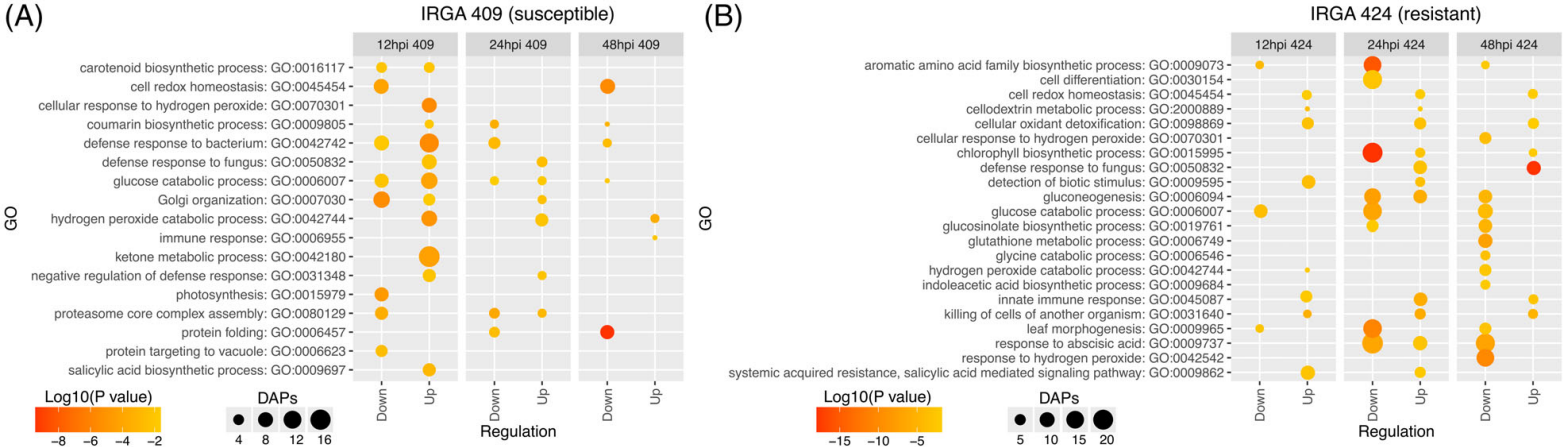
Gene ontology (GO) enrichment of differentially accumulated proteins in two rice genotypes under *M. oryzae* infection. (A) IRGA 409 and (B) IRGA 424.

GO term enrichment analysis of DAPs further aligned with these genotypes and time‐dependent differences (Figure 3). In the susceptible IRGA 409, proteins associated with “defense response to fungus” increased at 12 and 24 hpi, while processes related to cell redox homeostasis and protein folding decreased at 24 and 48 hpi; in contrast, the hydrogen peroxide catabolic process increased at all time points (12, 24, and 48 hpi) (Figure 3A). By comparison, IRGA 424 showed increased accumulation across all time points in pathways including cellular oxidant detoxification, cell redox homeostasis, innate immune response, and killing of cells of another organism (Figure 3B). In this resistant genotype, stress‐related pathways such as defense response to fungus, systemic acquired resistance (salicylic acid‐mediated), and detection of biotic stimulus involved a greater number of proteins with higher accumulation levels than in the susceptible genotype (Figure 3). Together with the PCA results and time‐resolved DAP profiles, these enrichment patterns indicate that IRGA 424 exhibits a faster, more coordinated, and more intense proteomic defense response to *M. oryzae*, whereas IRGA 409 shows a delayed response that appears less efficient.

Within the set of DAPs consistently accumulated across all three time points in the susceptible genotype, the protein Os01g0963600 (UniProt ID: Q94DL7), a member of the abscisic acid, stress and ripening (ASR) protein family and predicted to be encoded by the *OsASR6* gene, was of particular interest. Although ASR proteins are well known for roles in abiotic stress, their functions in plant resistance to biotic stimuli remain poorly understood, with only two studies reporting such a relationship to date (Guo, Chen, et al., 2022; Li et al., 2018). The most recent study, published by Guo, Chen, et al. (2022), reported that *OsASR6* is induced by pathogen and acts as a suppressor of resistance in rice by broadly modulating CIPK15 and WRKY45‐1 proteins mediated immunity, salicylic acid (SA) signaling, and redox homeostasis. Therefore, the accumulation of this protein after infection by *M. oryzae* in the susceptible genotype in our study may suggest a repression of plant resistance and the involvement of the ASR protein family in plant–fungal responses.

In contrast, the resistant genotype consistently accumulated stress‐related PR5 proteins across infection‐exposure time points, notably Q2QND6—Os12g0569500 (PR5, osmotin‐like) and Q2QLS7—Os12g0630200 (PR5, thaumatin‐like) (Figure 3H). PR5 proteins share homology with thaumatin‐like proteins (TLPs), suggesting similar functions (Islam et al., 2023). Previous studies have shown that overexpression of PR5 proteins in transgenic plants enhances disease resistance across various species (Chouhan et al., 2023; Fagoaga et al., 2001; Velazhahan & Muthukrishnan, 2003), and one of their key roles includes inhibiting pathogen signal transduction pathways, thereby increasing fungal cell susceptibility (El‐kereamy et al., 2011). Thus, the sustained accumulation of these PR5 proteins in IRGA 424 is likely associated with an enhanced capacity to face the infection.

### The phosphoproteome showed a more intense phospho‐regulation in the resistant rice genotype

Building on the premise that protein phosphorylation, like protein abundance, can serve as a biomarker and/or regulator of (a)biotic stress tolerance or susceptibility in crops (Fonseca de Lima et al., 2025), we investigated whether rice genotypes with contrasting resistance to blast disease exhibit shared or genotype‐specific phosphorylation patterns. To this end, we analyzed the phosphoproteomes of resistant and susceptible genotypes at 12, 24, and 48 hpi with *M. oryzae*, alongside kinase–substrate interaction predictions. Together, these analyses shed light on how *M. oryzae* infection dynamically modulates phosphorylation status and unveil key signaling networks associated with the plant's defense response.

**Figure 4:**
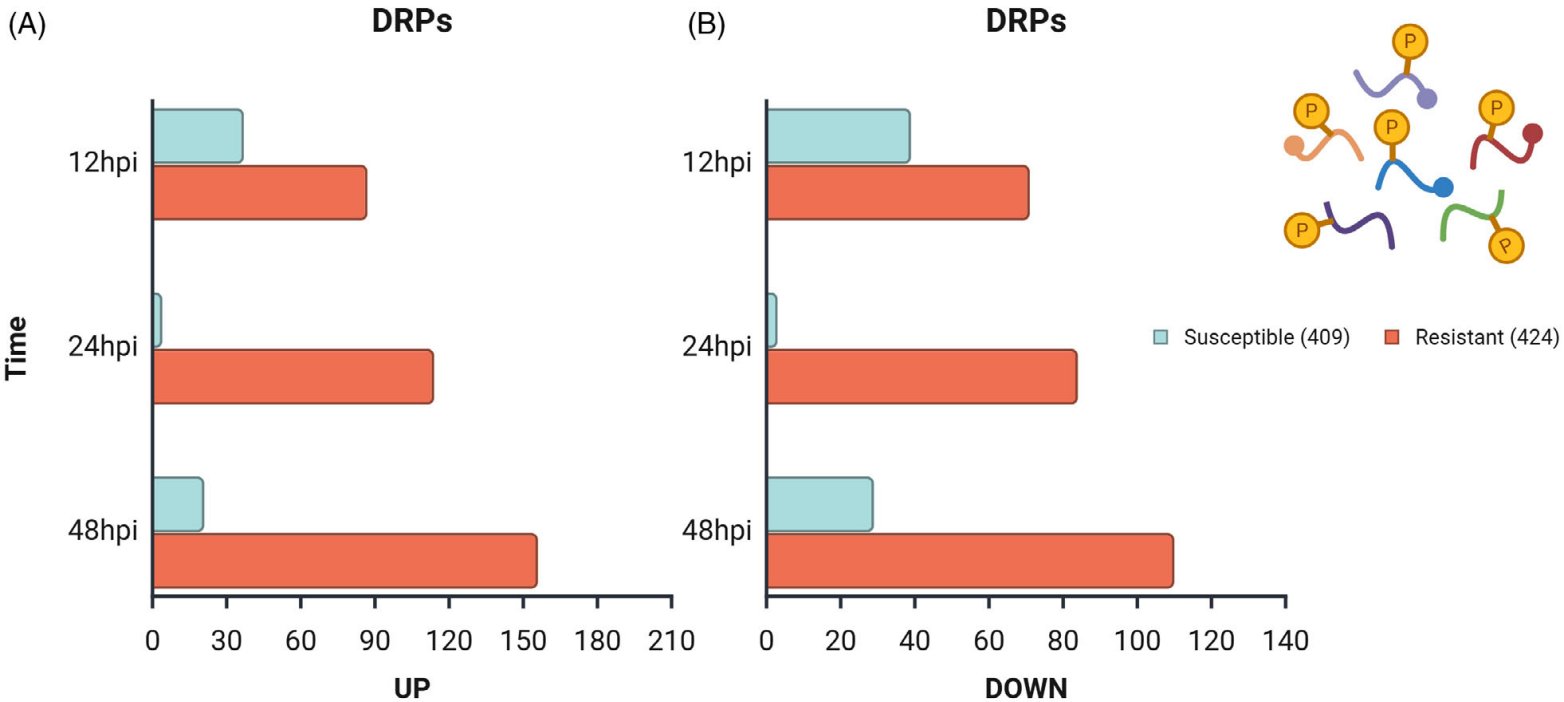
Differential regulation of phosphosites (DRPs) in rice genotypes IRGA 409 and IRGA 424 at 12, 24, and 48 h post‐inoculation (hpi). (A) Upregulated phosphosites. (B) Downregulated phosphosites.

**Figure 5:**
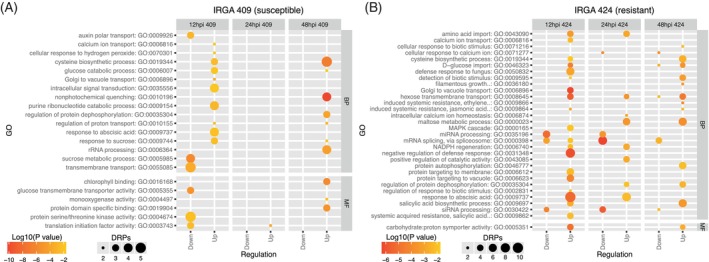
Gene Ontology (GO) terms associated with differentially regulated phosphoproteins in genotypes IRGA 409 (A) and IRGA 424 (B). The annotation of GO terms by protein was performed using GOMAP.

Across all samples, a total of 9653 phosphosites were quantified in the IRGA 409 genotype and 11 514 in the IRGA 424 genotype. In line with previous reports, including Nakagami et al. (2010), serine (S) was the most frequently phosphorylated amino acid, followed by threonine and tyrosine (Data S2 and Figure S2). The number of differentially regulated phosphosites (DRPs) also differed by genotype and time. In IRGA 409, 76, 7, and 50 DRPs were detected at 12, 24, and 48 hpi, respectively; of these, 37, 4, and 21 were upregulated (Figure 4A), while 39, 3, and 29 were downregulated (Figure 4B). By contrast, IRGA 424 exhibited higher DRP counts at all time points: 158, 198, and 266 DRPs at 12, 24, and 48 hpi, respectively, with 87, 114, and 156 upregulated (Figure 4A) and 71, 84, and 110 downregulated (Figure 4B). Notably, these values reflect significant changes in phosphorylation at specific sites between the compared groups and do not necessarily indicate corresponding changes in total protein abundance. To contextualize these site‐specific changes, we performed GO term enrichment of proteins harboring DRPs. In the susceptible genotype IRGA 409, enriched terms were mainly associated with auxin polar transport, cysteine biosynthetic process, and transmembrane transport (Figure 5A). In contrast, in the resistant genotype IRGA 424, enrichment was predominantly linked to stress defense mechanisms, including cellular response to biotic stimulus, defense response to fungus, detection of biotic stimulus, and salicylic acid biosynthetic process (Figure 5B).

In rice, as in several other species, protein phosphorylation plays a critical role in immune responses to phytopathogen infection (Hou et al., 2019). Pathogen‐triggered signals are relayed through kinase cascades and are regulated by kinases and phosphatases via phosphorylation and dephosphorylation events; these signals ultimately reach the nucleus, where disease resistance‐related proteins are directly or indirectly modified to activate immune defenses (Hou et al., 2015). Within this framework, the present study revealed marked differences between genotypes: the resistant IRGA 424 genotype displayed a higher number of differentially regulated phosphorylation sites and phosphoproteins than the susceptible IRGA 409 at all infection time points (Figure 4), suggesting more intense post‐translational regulation through phosphorylation. Similar differences between resistant and susceptible rice cultivars in response to *M. oryzae* have been reported in phosphoproteomic analyses using two‐dimensional electrophoresis (Li et al., 2015), indicating that such modulation may represent a general proteomic signature of resistance. Furthermore, the [S_P] motif showed the highest frequency, especially in the resistant genotype (Figures S3 and S4), and is commonly recognized as a substrate for RLKs, CDPKs, and MAPKs (Wang et al., 2017).

### Kinase–substrate prediction: Divergent predicted substrate responses as potential drivers of resistance versus susceptibility

To identify kinases and putative substrates potentially involved in regulating resistance or susceptibility during the early stages of *M. oryzae* infection in rice plants, we applied a kinase–substrate prediction approach based on differentially regulated phosphosites (DRPs) from the IRGA 424 (resistant) and IRGA 409 (susceptible) phosphoproteomes. To add contextual validation, an additional co‐localization analysis was performed to examine the subcellular localization of these kinases and their putative substrates; only predicted kinase–substrate relationships with compatible subcellular localization are discussed below.

Here, predicted substrates are defined as phosphorylated proteins whose phosphosites were computationally assigned to specific kinases using a motif‐based approach. A total of 133 DRPs within 96 proteins were identified across all time points in the susceptible genotype (IRGA 409), of which 82 were predicted substrates of CAMK, 71 of SnRK2, 49 of CK2, 12 of MAPK, 11 of CDPK, 10 of AGC, and 9 of RLK. In contrast, 622 DRPs within 562 proteins were identified in the resistant genotype (IRGA 424), including 379 predicted substrates of CAMK, 335 of SnRK2, 255 of CK2, 44 of CDPK, 33 of MAPK, and 31 each of AGC and RLK. Note that the total number of predicted kinase–substrate associations exceeds the number of DR phosphosites because individual phosphosites may be predicted as substrates of multiple kinases.

At 12 hpi, CAMK, CK2, and SnRK2 accounted for the largest number of predicted substrates in both genotypes. Among these, a CK2‐predicted substrate detected exclusively in the resistant phosphoproteome, Os12t0407500‐01 protein (*OsPTEN*; UniProt: Q0INR2), displayed a phosphosite fold change (FC) of 4.18. This finding aligns with recent work showing that *OsPTEN*, a PTEN‐family phosphatase, positively regulates rice immunity against *M. oryzae* by dephosphorylating *OsATL32*, an E3 ubiquitin ligase that negatively modulates defense. Overexpression of *OsPTEN* resulted in smaller lesions and reduced fungal proliferation compared to the wild type, whereas *Ospten* loss‐of‐function plants were more susceptible, supporting *OsPTEN* as a positive regulator of blast resistance (Yan et al., 2024).

**Figure 6:**
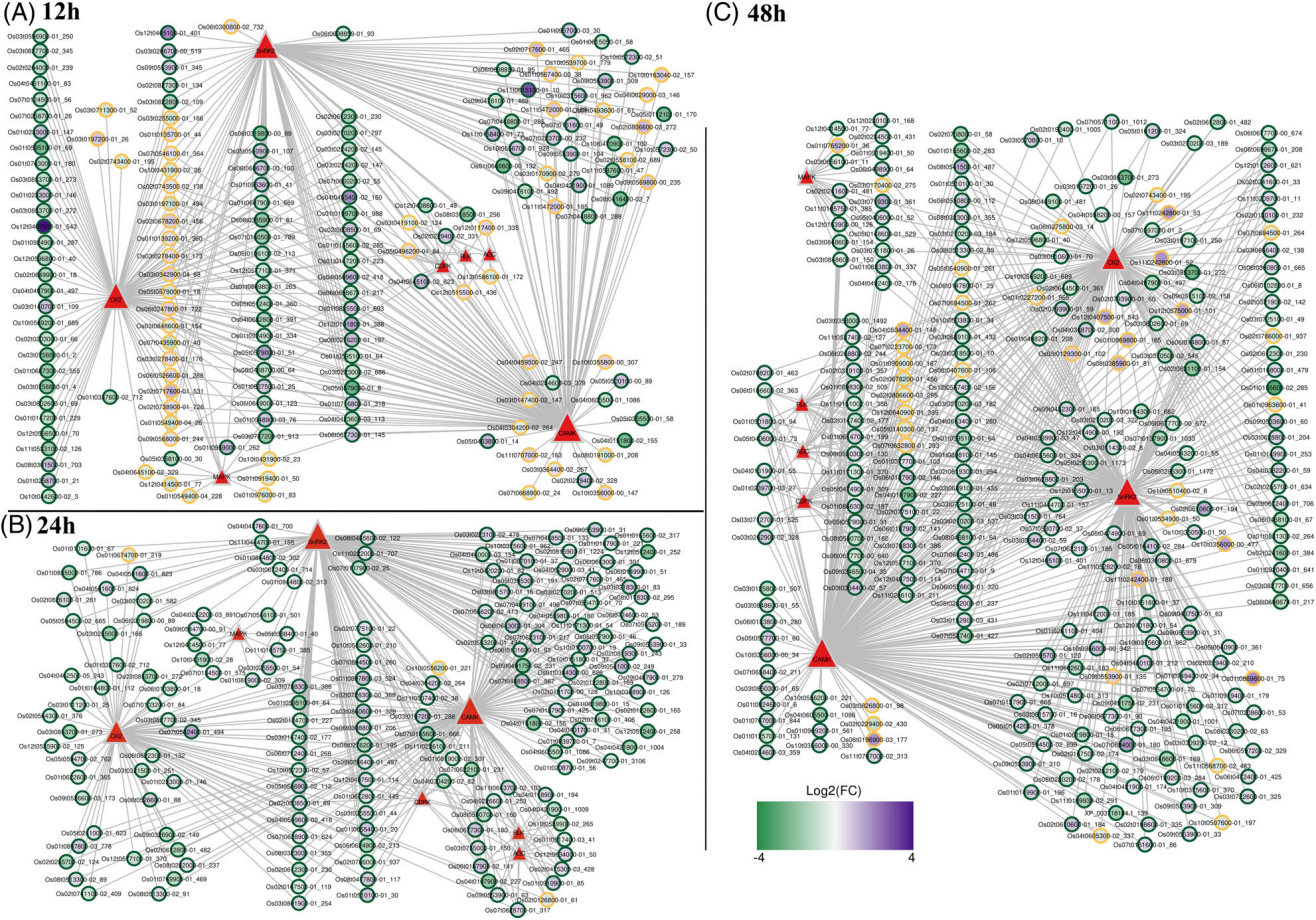
Predicted kinase–substrate interactions in the phosphoproteomic datasets of IRGA 409 (susceptible) and IRGA 424 (resistant) at 12 (A), 24 (B), and 48 (C) hours after *Magnaporthe oryzae* inoculation. Green circles represent proteins (substrate predicted) from the resistant genotype, while yellow circles represent those from the susceptible genotype. Red triangles indicate the predicted kinases. The log2(FC) reflects the differential regulation of phosphorylation sites, and node size (red triangles) represents degree centrality.

**Figure 7:**
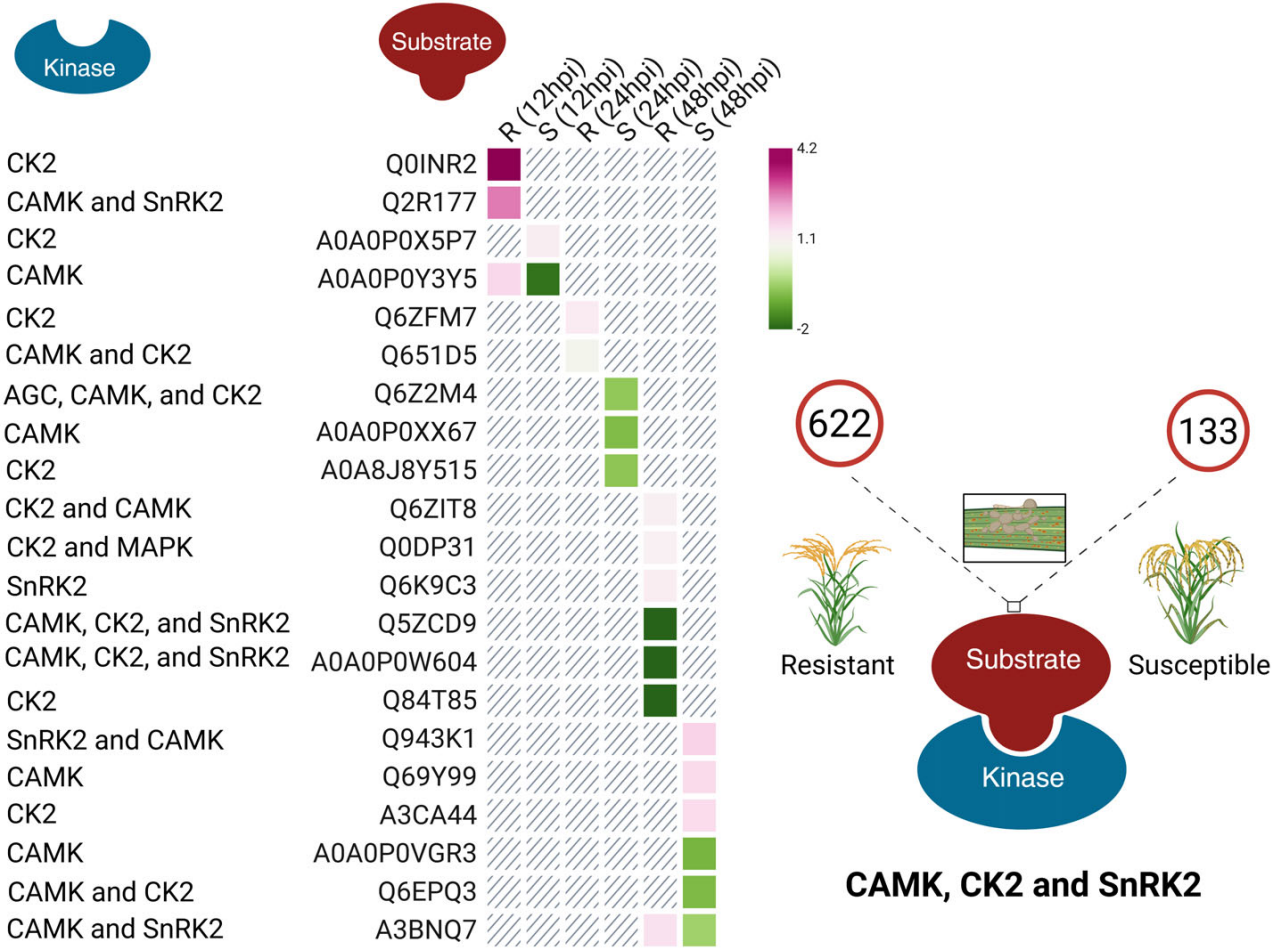
Illustrative summary of the main kinase–substrate predictions in IRGA 409 (susceptible) and IRGA 424 (resistant) at 12, 24, and 48 h post *Magnaporthe oryzae* inoculation. CAMK, CK2, and SnRK2 were the kinases associated with the highest number of predicted substrates in both rice genotypes at all time points. A total of 133 differentially regulated phosphosites (DRPs) were identified across all time points in the susceptible genotype (IRGA 409), and 622 DRPs were identified in the resistant genotype (IRGA 424). Among these, some substrates, defined here as phosphorylated proteins containing phosphosites computationally linked to specific kinases using a motif‐based prediction strategy, were differentially or exclusively regulated between genotypes and are shown in the heatmap. Magenta–purple colors indicate upregulated substrates, and green colors indicate downregulated substrates. The absence of color indicates that the substrate was not differentially regulated in the genotype and/or at the time point. R denotes the resistant genotype and S the susceptible. Predicted kinases for the displayed substrates are listed to the left of the heatmap. Created in BioRender. Auler, P. (2025) https://BioRender.com/12gk2te.

Another predicted substrate, Os11t0615100‐01_10 (UniProt: Q2R177), was differentially regulated exclusively in the resistant genotype and co‐localized with both CAMK and SnRK2, exhibiting a phosphosite FC of 2.87. By contrast, only one predicted substrate was exclusively regulated in the susceptible genotype at 12 hpi: Os07t0435900‐01_40 (UniProt: A0A0P0X5P7; phosphosite FC of 1.3). Finally, a CAMK‐predicted substrate in the phosphoprotein Os11t0587600‐01_47 (UniProt: A0A0P0Y3Y5) was upregulated in the susceptible genotype (FC = 1.79) but downregulated in the resistant genotype (FC = −1.80) (Figures 6A and 7). According to UniProt, this protein belongs to the ATP‐binding cassette (ABC) transporter G family, member 34. Consistent with a potential role in defense signaling, plant ABCG transporters primarily mediate the secretion of secondary metabolites and defense‐related hormones, which are essential for countering pathogen attack (Dhara & Raichaudhuri, 2021).

At 24 h hpi, CAMK, CK2, and SnRK2 again accounted for the largest share of predicted substrates in both genotypes. Among these, a differentially regulated substrate detected exclusively in the resistant genotype mapped to the phosphoprotein Os09t0541000‐02_249 (UniProt: Q651D5), predicted to be targeted and co‐localized with CAMK, showed a phosphosite FC of 0.99. Additionally, MAPK, RLK, and CDPK were associated exclusively with DRPs in the resistant genotype, indicating that none of the DRPs detected in the susceptible genotype at 24 hpi were predicted targets of these kinase families (Figures 6B and 7).

In contrast, two DRPs with predicted kinase associations were identified exclusively in the susceptible genotype at 24 hpi, both of which contained downregulated phosphosites: Os02t0126800‐01_61 (UniProt: Q6Z2M4; FC = −0.56) and Os10t0556200‐01 (UniProt: A0A0P0XX67; FC = −0.75), associated and co‐localized exclusively with CAMK. The Q6Z2M4 protein encodes a component of the SNARE (SNAP) receptor complex. In rice, *OsSNAP32* is induced upon infection with *M. oryzae*, and its overexpression enhances resistance in otherwise susceptible genotypes. In contrast, RNAi‐mediated silencing in a resistant background increases lesion formation, shifting the phenotype toward susceptibility (Luo et al., 2016). Thus, both gain and loss‐of‐function evidence support a positive role for SNAP family members in blast resistance. The reduction in phosphorylation of this protein exclusively in the susceptible genotype is consistent with and may contribute to the susceptibility observed in the IRGA 409 genotype.

At 48 hpi, CAMK, CK2, and SnRK2 remained the kinase families associated with the highest number of predicted substrates in both genotypes. Among the substrates exclusively regulated in the resistant genotype, phosphosites on Os08t0541500‐01_487 (UniProt accession: Q6ZIT8; predicted targets: CK2 and CAMK) increased by 1.26‐fold. A third substrate, Os02t0610600‐01_194 (UniProt accession: Q6K9C3), also exclusively differentially regulated in the resistant genotype and predicted to be targeted by SnRK2, showed an FC of 1.34. Among the exclusively regulated substrates with downregulated phosphosites in the resistant genotype, two exhibited pronounced decreases (FC < −2): Os01t0155600‐02_283 (UniProt accession: Q5ZCD9), predicted targets of CAMK, CK2, and SnRK2; and Os03t0853700‐01_272 (UniProt accession: Q84T85), predicted to be targeted exclusively by CK2.

In the susceptible genotype, two were exclusively downregulated: Os02t0229400‐02_430 (UniProt accession: A0A0P0VGR3; FC = −0.86) and Os02t0678200‐01_456 (UniProt accession: Q6EPQ3; FC = −0.78), both of which were predicted CAMK targets. Finally, a predicted CAMK substrate, Os07t0694000‐01_180 (UniProt accession: A3BNQ7), showed opposite regulation between genotypes: the phosphosite was upregulated in the resistant genotype but downregulated in the susceptible genotype (Figures 6C and 7). A3BNQ7, annotated as a phosphoinositide‐specific phospholipase C (PI‐PLC), plays a central role in plant innate immunity against fungi and other pathogens (Abd‐El‐Haliem & Joosten, 2017). Acting as an early signaling hub, PI‐PLC is rapidly activated upon pathogen perception by immune receptors, triggering the hydrolysis of phosphatidylinositol 4,5‐bisphosphate and initiating downstream calcium mobilization and signaling cascades that culminate in robust, multilayered defense responses (Vossen et al., 2010). In our dataset, A3BNQ7 showed increased phosphosite abundance in the resistant genotype (FC = 1.63) but decreased abundance in the susceptible genotype (FC = −0.40). In light of evidence that rapid PI‐PLC activation enhances disease resistance, this contrasting regulation further supports a contributory role of PI‐PLC to resistance against *M. oryzae*.

Overall, the resistant genotype displayed a substantially greater number of differentially regulated phosphosites and predicted kinase–substrate associations than the susceptible genotype, indicating a more complex and dynamic phosphorylation‐driven signaling landscape. Consistently across all infection time points, CAMK, CK2, and SnRK2 were associated with the largest sets of predicted substrates in both genotypes (Figures 6 and 7). In plants, CAMK typically refers to Ca^2+^/calmodulin‐dependent kinases (Chen et al., 2021), whereas SnRK2s constitute the core of the abscisic acid signaling pathway and strongly modulate stomatal immunity and phytohormone crosstalk (Shinozawa et al., 2019). Protein kinase CK2 (formerly casein kinase II) is a ubiquitous serine/threonine kinase conserved across eukaryotes, with more than 300 substrates spanning diverse cellular processes (Mulekar & Huq, 2014). Although motif matching alone does not guarantee kinase–substrate specificity, the predicted associations are supported here by the overlap in subcellular localization and the established involvement of these kinase families in the corresponding defense pathways. The identification of predicted substrates that are exclusively present in a specific genotype (resistant or susceptible), as observed here, is likely to be critical for a better understanding of the differences in plant immunity responses to infection. Such substrates may represent key regulatory nodes that distinguish between resistance and susceptibility mechanisms.

### Acetylome landscape

Protein acetylation can also serve as an indicator of pathogen‐mediated regulation of plant defense responses (Walley et al., 2018). Therefore, acetylation sites were quantified, and the main differentially acetylated proteins associated with blast disease or other biotic stresses were identified based on Trait Ontology (TO) and Gene Ontology (GO) annotations. To the best of our knowledge, this is the first study comparing acetylation sites and acetylated proteins across contrasting rice genotypes under *M. oryzae* infection. A previous study analyzed the acetylome of a single rice genotype overexpressing an *M. oryzae*‐responsive gene (*MSP1*) (Lee et al., 2023), in which a lower number of acetylation sites and acetylated proteins were identified compared to what was found in the present work.

**Figure 8:**
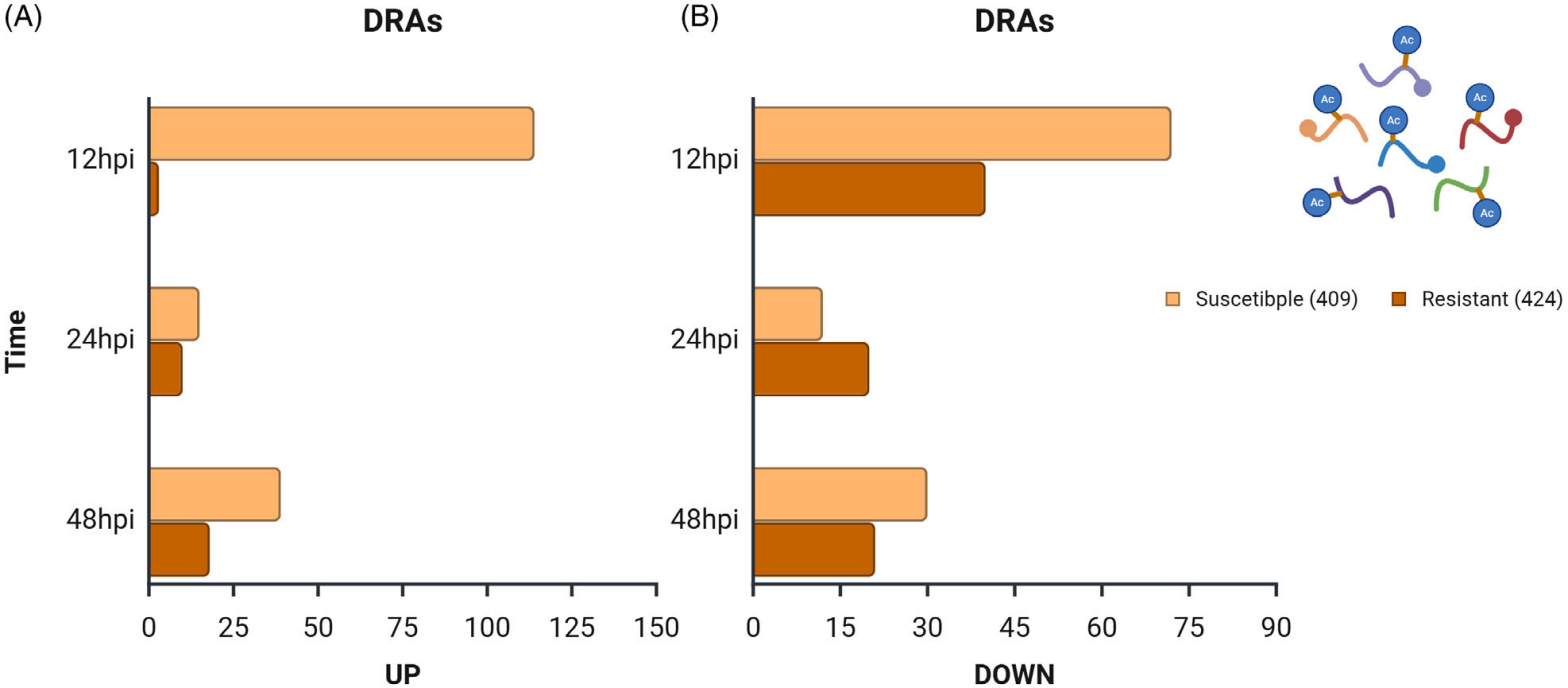
Differential regulation of acetylsites (DRAs) in rice genotypes IRGA 409 and IRGA 424 at 12, 24, and 48 h post‐inoculation (hpi). (A) Number of upregulated acetylsites. (B) Number of downregulated acetylsites.

**Figure 9:**
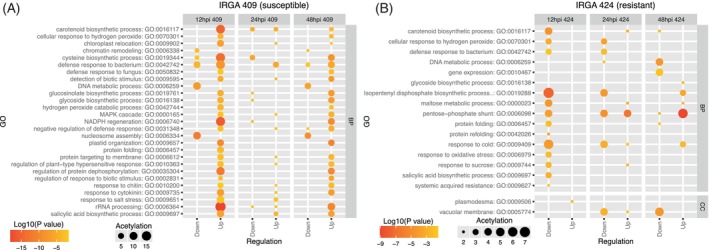
GO enrichment of differentially acetylated proteins in two rice genotypes under *M. oryzae* infection. GO enrichment analysis of acetylated proteins quantified in the IRGA 409 (A) and IRGA 424 (B) genotypes at 12, 24, and 48 h post‐inoculation (hpi).

In both genotypes, the highest number of acetylsites was quantified at 12 hpi, the earliest phase of the infection (Data S3 and Figure S5). The number of DRAs in the IRGA 409 genotype was 186, 27, and 69 at 12, 24, and 48 hpi, respectively. Of these, 114, 15, and 39 were upregulated (Figure 8A) and 72, 12, and 30 were downregulated (Figure 8B). In IRGA 424, the number of DRAs was 41, 35, and 39 at 12, 24, and 48 hpi, respectively. Of these, 3, 10, and 18 were upregulated (Figure 8A) and 40, 25, and 21 were downregulated (Figure 8B). The lack of comparative data makes it challenging to interpret the observed responses; however, it highlights the importance of this acetylome generated here under these specific conditions as a valuable resource for more targeted studies. Interestingly, in contrast to the observations in the global proteome and phosphoproteome analyses, the resistant genotype exhibited fewer DRAs at 12 and 48 hpi than the susceptible genotype (Figure 9D). Similarly to the hypothesis generated by the phosphoproteome analysis, the differential increase in acetylation sites may indicate a protein signature of susceptibility to blast disease in rice; however, further studies are needed to confirm these hypotheses.

According to Nallamilli et al. (2014), although a clear consensus sequence was not identified, there is a noticeable sequence bias around lysine acetylation sites. The authors reported that amino acid frequency analysis within a ±6 position window from the acetylated lysine in rice revealed that the KacS motif, where serine is located at the +1 position, ranked among the top four most frequent patterns, accounting for 6.9% of occurrences. Additionally, glycine at the −1 position was the second most frequent residue, present in 11.1% of the cases. These motifs were also identified in both genotypes in our study using the differentially regulated acetylated proteins (DRAPs), as shown in the Figure S6.

### Acetylated proteins related to stress responses and plant–pathogen interactions

Since the susceptible genotype generally exhibited a higher number of DRAs than the resistant genotype (Figure 8), we proceeded to protein‐level analyses to investigate potential associations between these proteins and blast disease. To identify the DRAPs associated with blast disease or other biotic stresses, they were uploaded to the Rice Annotation Project Database (RAP‐DB) for functional characterization. This database classifies the identified proteins using ontologies, such as the TO and GO. Some DRAPs were found to be associated with blast disease, stress responses, and plant–pathogen interactions, and these proteins are presented in Table 1 (IRGA 409) and 2 (IRGA 424). The complete tables are available in Data S4–S15.

**Table 1:** Acetyl‐proteins related to stress responses and plant–pathogen interactions with differential acetylation sites between mock and pathogen‐infected IRGA 409 (susceptible genotype) plants at 12, 24, and 48 h post‐inoculation (hpi)

DRAPs_IRGA 409	Time	Description	Trait Ontology (TO) or Gene Ontology (GO)	Acetyl site position	Log 2FC
Os04t0602100	12 hpi	Plant ascorbate peroxidase protein	Cellular response to oxidative stress (GO:0004601)	K113	0.66
Os01t0896500	12 hpi	Rhodanese‐like protein	Cellular response to calcium ion (GO:0071277)	K201	0.64
Os02t0257300	12 hpi	Thylakoid rhodanese‐like protein	**Blast disease** (TO:0000074)	K187	0.85
Os02t0553200	12 hpi	Thylakoid membrane‐bound ascorbate peroxidase	Bacterial blight disease resistance (TO:0000175)	K267	0.87
Os01t0639900	12 hpi	Beta‐carbonic anhydrase	**Blast disease** (TO:0000074)	K148	−0.62
Os02t0787800	12 hpi	Diacylglycerol kinase	**Blast disease** (TO:0000074)	K431	−0.61
Os08t0379400	12 hpi	Similar to Quinone oxidoreductase‐like protein	**Blast disease** (TO:0000074), bacterial blight disease resistance (TO:0000175)	K259	−0.79
Os03t0710800	24 hpi	Member of the 14–3‐3 protein family	Abscisic acid sensitivity (TO:0000615)	K159	−0.41
Os11t0707000	24 hpi	Small isoform of RuBisCO activase	**Blast disease** (TO:0000074)	K308	0.80
Os07t0152900	24 hpi	Glycolate oxidase	Stomatal conductance (TO:0000522)	K218	0.65
Os01t0896500	24 hpi	Rhodanese‐like domain	Regulation of stomatal closure (GO:0090333)	K475	−0.55
Os03t0131200	24 hpi	Catalase C	Bacterial blight disease resistance (TO:0000175) **blast disease** (TO:0000074)	K5	−0.54
Os01t0639900	48 hpi	beta‐carbonic anhydrase	**Blast disease** (TO:0000074)	K92	0.70
Os02t0537700	48 hpi	2‐Cys peroxiredoxin	Oxidative stress (TO:0002657)	K155	0.52
Os06t0264200	48 hpi	CONSTANS‐like protein	Jasmonic acid sensitivity (TO:0000172)	K15	−0.58
Os07t0100200	48 hpi	Pyridoxal phosphate synthase	Bacterial leaf streak disease resistance (TO:0000203)	K106	−0.62

*Note*: Proteins already associated with blast disease according to Trait Ontology are highlighted in bold.

Acetylation of Catalase C (Os03t0131200) decreased in both genotypes; however, the temporal and site‐specific patterns diverged, with hypoacetylation detected at 24 hpi in the susceptible IRGA 409 and at 48 hpi in the resistant IRGA 424. Moreover, the affected sites were distinct, residue 5 at the N terminus in IRGA 409 (Table 1) versus residue 401 in IRGA 424 (Table 2). These observations suggest that genotype‐specific regulatory mechanisms may modulate activation, protein–protein interactions, or subcellular localization. Although functional data regarding these site‐specific modifications are currently lacking, previous studies have already reported that Catalase C plays a prominent role over other catalases during fungal infection in sugarcane (Gujjar et al., 2025).

**Table 2:** Acetyl‐proteins related to stress responses and plant–pathogen interactions with differential acetylation sites between mock and pathogen‐infected in the genotype IRGA 424 (resistant) in 12, 24, and 48 hpi

DRAPs_IRGA 424	Time	Description	Trait Ontology (TO) or Gene Ontology (GO)	Acetyl site position	Log2FC
Os02t0815500	12 hpi	S‐nitrosoglutathione reductase	Salt tolerance (TO:0006001)	K238	1.19
Os11t0242800	12 hpi	Light‐dependent rice blast resistance	**Blast disease** (TO:0000074)	K225	0.92
Os04t0448900	12 hpi	Zeaxanthin epoxidase	Viral disease resistance (TO:0000148), bacterial leaf streak disease resistance (TO:0000203)	K406	−0.60
Os11t0707000	24 hpi	Small isoform of RuBisCO activase	**Blast disease** (TO:0000074)	K80	0.51
Os01t0896500	24 hpi	Rhodanese‐like protein.	Cellular response to calcium ion (GO:0071277)	K467	0.62
Os03t0710800	24 hpi	Member of the 14–3‐3 protein family	Abscisic acid sensitivity (TO:0000615)	K131	−0.80
Os11t0242400	24 hpi	photosystem II protein 33	**Blast disease** (TO:0000074)	K231	−0.67
Os04t0602100	24 hpi	Plant ascorbate peroxidase protein	Response to oxidative stress (GO:0006979)	K136	−0.53
Os01t0574600	48 hpi	Glyoxylate reductase	Oxidoreductase activity (GO:0016491)	K284	0.54
Os03t0710800	48 hpi	Member of the 14–3‐3 protein family	Abscisic acid sensitivity (TO:0000615)	K131	0.57
Os03t0131200	48 hpi	Catalase C	**Blast disease** (TO:0000074)	K401	−0.54
Os03t0297100	48 hpi	40S ribosomal protein S7	Sheath blight disease resistance (TO:0000255)	K107	−0.67

*Note*: Proteins already associated with blast disease according to Trait Ontology are highlighted in bold.

The rhodanese‐like domain–containing protein STR10 (Os01t0896500) also displayed differential acetylation across sites, time points, and genotypes. Specifically, in susceptible IRGA 409, K201 was hyperacetylated at 12 hpi, and K475 was hypoacetylated at 24 hpi. However, in the resistant IRGA 424, K467 was hyperacetylated at 24 hpi. Rhodanese‐like proteins have been implicated in the detoxification of ROS during stress. Consistently, in Arabidopsis plants, a rhodanese‐like domain–containing protein interacts with cytosolic hydroxymethyl dihydropterin pyrophosphokinase (AT1G69190), an enzyme linked to oxidative stress responses (Bhattacharjee et al., 2020). The observed acetylation signatures, distinct in timing and residue, are therefore compatible with a model in which STR10 fine‐tunes ROS detoxification during *M. oryzae* infection, with acetylation state modulating protein activity, interactions, or subcellular distribution in a genotype‐specific manner.

GF14‐F (UniProt Q06967), a 14‐3‐3 family member implicated in regulating stomatal aperture to limit pathogen entry (Kaundal et al., 2017), displayed site, time, and genotype‐dependent acetylation changes. At 24 hpi, acetylation decreased in both genotypes, specifically at K159 in IRGA 409 (Table 1) and at K131 in IRGA 424 (Table 2). By 48 hpi, the resistant genotype exhibited hyperacetylation at K131, the same site that was previously hypoacetylated at 24 hpi. Although 14‐3‐3 proteins are classically recognized as phospho‐binding adaptors, acetylation of 14‐3‐3s has also been reported in Arabidopsis (Guo, Chai, et al., 2022), positioning them as crosstalk nodes between acetylation and phosphorylation. Consistent with predictive models, lysine deacetylation under stress may enhance 14‐3‐3 interactions with phosphorylated targets (Guo, Chai, et al., 2022), providing a potential mechanistic context for the site and time‐specific dynamics observed for GF14‐F. Similarly, a dynamic immune acetyl‐proteomics study in maize infected by *Puccinia polysora* showed that a 14‐3‐3 protein was slightly upregulated in the resistant genotype but downregulated in the susceptible one (Guo, Ma, et al., 2022), and the authors also identified K131 as an acetylation site, further supporting our findings.

GO term enrichment analysis of acetylated proteins revealed enrichment across a larger set of pathways in genotype IRGA 409 than in IRGA 424, as expected given the higher number of DRAs in this genotype, highlighting the response to chitin, regulation of plant‐type hypersensitive response, and protein targeting to membrane pathways (Figure 9A). In the IRGA 424 genotype, the majority of enriched pathways were related to protein folding, defense response to bacterium, response to sucrose, and cellular response to hydrogen peroxide (Figure 9B).

### Relationship between differentially accumulated proteins and PTMs

To assess whether differences in protein accumulation are associated with phosphorylation and/or acetylation, we examined the DAPs across genotypes and infection time points that exhibited PTMs. Specifically, we compared the DAP lists with sets of differentially regulated phosphoproteins and acetylated proteins for each genotype at all exposure times. Additionally, phosphosite conservation analysis of some of these proteins was performed to support the functional relevance of the prioritized PTM sites.

**Figure 10:**
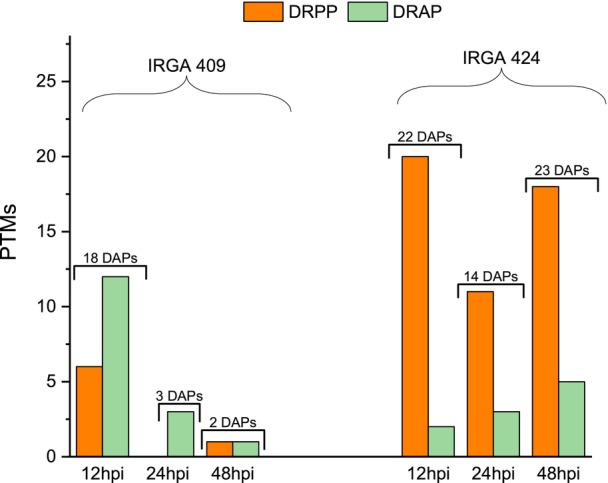
Differentially accumulated proteins (DAPs) identified among differentially regulated phosphoproteins (DRPP) and acetyl‐proteins (DRAP) in the IRGA 409 (susceptible) and IRGA 424 (resistant) genotypes at 12, 24, and 48 h post‐inoculation (hpi). Above the bars are the numbers of the DAPs that presented DRPP and/or DRAP at each time point. DPRR is represented in orange and DRAP in green.

In the susceptible genotype IRGA 409, 23 DAPs carried phospho‐ and/or acetyl‐modifications, whereas 59 DAPs presented PTMs in the resistant genotype (IRGA 424). Notably, in line with the previous results, DAPs in IRGA 409 tended to show relatively more acetyl‐modifications, whereas DAPs in the resistant genotype displayed more phospho modifications. In IRGA 409, 16 DAPs were modified by acetylation and 6 by phosphorylation across all time points. In IRGA 409, 49 DAPs exhibited phosphorylation and 10 acetylation (Figure 10 and Data S18).

Another important relationship was observed. Of the 29 up‐accumulated proteins in the resistant genotype, 25 were up‐phosphorylated, and only 4 up‐accumulated proteins were down‐phosphorylated. Similarly, among the 20 down‐accumulated proteins in this genotype, all were down‐phosphorylated. The same pattern was observed in the susceptible genotype, where three proteins were both up‐accumulated and up‐phosphorylated, and three were down‐accumulated and down‐phosphorylated. Among DAPs with differential acetylation in the resistant genotype, four were up‐accumulated; of these, three were up‐acetylated and one was down‐acetylated. In the susceptible genotype, eight proteins were up‐accumulated, of which seven were up‐acetylated, while eight were down‐accumulated, of which five were up‐acetylated. Conversely, three down‐accumulated proteins exhibited increased acetylation (Data S18).

Although most commonly associated with other cellular events, PTMs can regulate protein abundance both directly and indirectly. Broadly, PTMs tune the flux between synthesis and degradation, and they modulate subcellular localization, complex assembly, and solubility, factors that alter protein half‐life and even translation rates, thereby shaping the observed abundance (Suskiewicz, 2024). Protein abundance increases/decreases and PTMs can co‐vary, for example, when a treatment activates a pathway that simultaneously induces or represses expression and triggers PTMs that stabilize the protein. While peaks in phosphorylation/acetylation typically precede changes in abundance, the lag can range from minutes to a few hours (Hammarén et al., 2022). Consequently, changes detected at 12, 24, or 48 hpi may arise from modifications that occurred prior to sampling. In our data, only one DAP bearing a previously identified PTM was detected at the earlier time point, suggesting response times shorter than 12 h (*lag* time) for most targets. Thus, although these associations are shown here, they do not imply immediate causality, as PTMs are rapid events that often precede abundance shifts.

Among these proteins, Os03t0197200‐01, annotated as a major facilitator superfamily (MFS) sugar transporter, showed increased phosphorylation and accumulated at 24 and 48 hpi. Notably, phosphorylation at 24 hpi mapped to S288, which may be associated with its accumulation at 24 and 48 hpi, whereas phosphorylation at 48 hpi occurred at serine 26 (S26). Also, this regulation was observed only in the resistant genotype (IRGA 424). Consistent with this, (Liu et al., 2021) reported that CgMFS1 is essential for sugar transport, oxidative stress resistance, and, consequently, the pathogenicity of *Colletotrichum gloeosporioides* toward plants such as *Hevea brasiliensis*. Thus, the phospho‐regulation and increased abundance of this transporter observed in our study may reflect regulatory mechanisms underlying resistance to blast disease.

Among the PTM‐modified DAPs, two chaperones (Os04t0107900‐02 and Os02t0758000‐01) were decreased in abundance and hypophosphorylated in the resistant genotype at 48 hpi. These stress‐responsive proteins are members of the heat shock protein (HSP) family. Hsp90 plays a critical role in fungal pathogenicity by regulating the folding and stability of key virulence factors; its ability to act as a molecular “transistor” enables rapid signaling changes required for successful infection (Neves‐da‐Rocha et al., 2023). It is therefore biologically plausible that phosphorylation changes could be associated with shifts in the abundance of these proteins, although reports of HSP hypophosphorylation in plants remain largely indirect.

A common approach for predicting functional phosphosites is conservation‐based sequence analysis (Kalyuzhnyy et al., 2025). Distinguishing sites that are functionally relevant becomes challenging due to the presence of multiple phosphorylated residues within the same protein and the ability of kinases to target several sites (Yaron‐Barir et al., 2024). Site conservation analysis therefore remains an effective strategy for identifying potentially functional phosphosites. To this end, we performed a conservation analysis of 39 phosphoregulated DAPs using orthologs from *Zea mays*, *Arabidopsis thaliana*, and *Sorghum bicolor*. Among these, 16 showed 100% conservation, 12 exhibited ≥50% conservation, and 11 displayed <50% conservation (Figure S7). Although functional phosphosites are generally expected to be highly conserved, since mutations disrupting phosphorylation could impair protein function and be selected against (Budovskaya et al., 2005), low conservation in some cases may reflect limitations of the available orthologs rather than an absence of functional relevance.

## CONCLUSION

**Figure 11:**
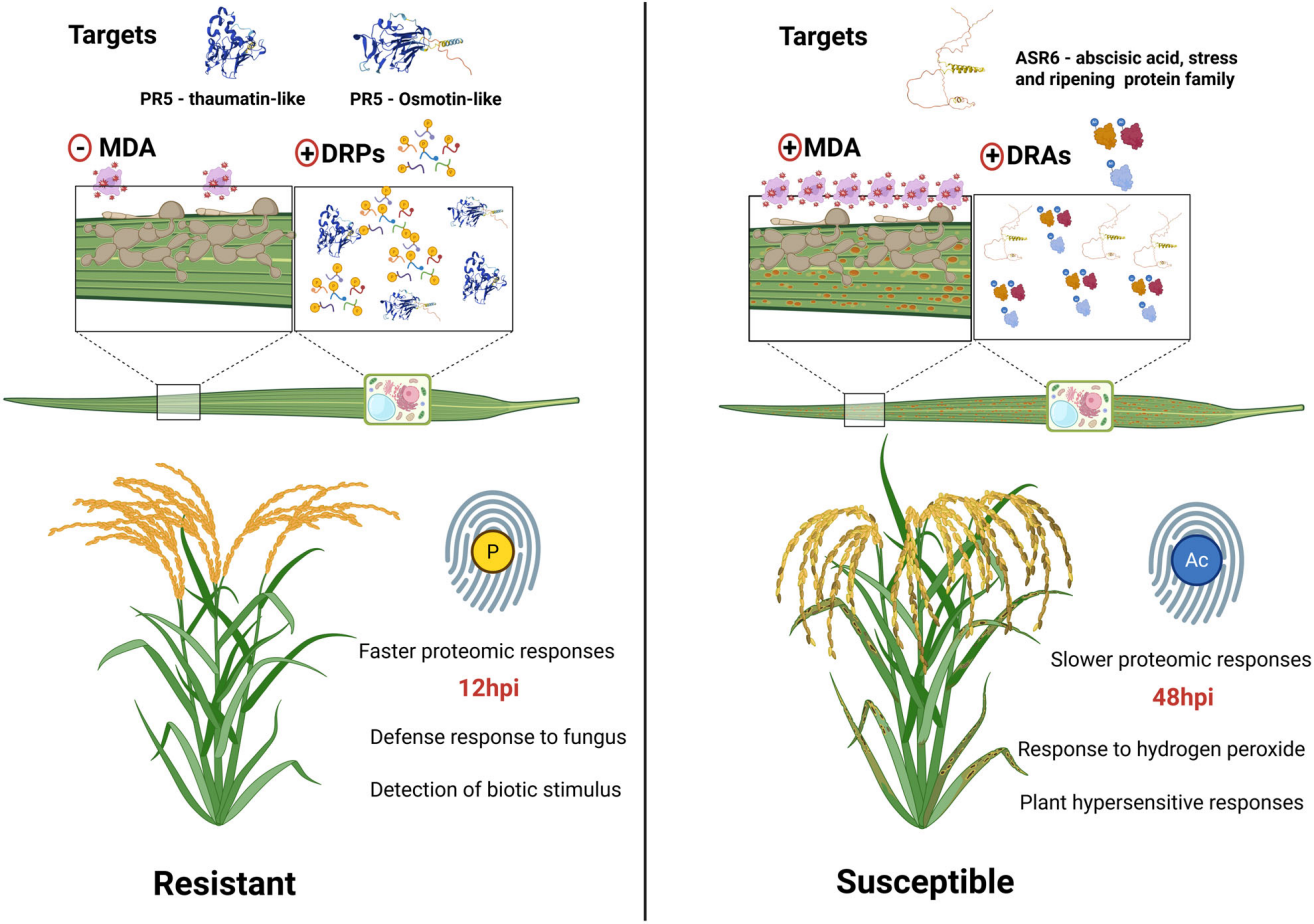
Overview of the main differences between the rice genotypes resistant (IRGA 424) and susceptible (IRGA 409) to blast disease identified in this study. At the biochemical level, the susceptible genotype exhibited higher levels of membrane lipid peroxidation (+MDA) compared with the resistant genotype (−MDA). The global proteome analysis identified potential targets for future investigation, highlighting pathogenesis‐related 5 proteins (thaumatin and osmotin‐like) in the resistant genotype and the protein ASR6 (an abscisic acid, stress, and ripening protein family member) in the susceptible genotype. Regarding the post‐translational modifications analyzed, the resistant genotype showed higher levels of regulation in the phosphoproteome (+DRPs), whereas the susceptible genotype did so in the acetylome (+DRAs). In addition, principal component analyses revealed that the resistant genotype formed distinct proteomic clusters between mock and infected plants as early as 12 hpi (faster proteomic responses), whereas in the susceptible genotype this separation only became evident at 48 hpi (slower proteomic responses). In the functional enrichment analyses of the proteomes, the resistant genotype showed consistent enrichment of pathways related to defense against fungi and detection of biotic stimuli, whereas the susceptible genotype showed enrichment of pathways associated with oxidative stress, such as responses to hydrogen peroxide and hypersensitivity. The fingerprints shown in the figure suggest a potential differential signature between these genotypes with respect to blast disease, in which the resistant genotype exhibits an increased phospho‐regulatory signature, while the susceptible genotype exhibits an acetyl‐regulatory signature. Created in BioRender. Auler, P. (2025) license number NL28YWBNOH.

The global proteome and PTMs analyses conducted in this study highlight the complexity and specificity of molecular responses in rice genotypes with contrasting resistance to *M. oryzae* infection. The divergent profiles of protein accumulation and modification between the genotypes raise an important question: Are there a few key molecular targets that confer enhanced resistance in plants? Given the intricate and interdependent nature of protein responses, manipulating individual genes may not be sufficient to achieve robust resistance. Nonetheless, the distinct molecular signatures uncovered here (Figure 11) provide valuable insights into the cellular mechanisms that underpin biotic stress responses.

Our study did not include pre‐inoculation (0 h) samples. We therefore quantified infection‐induced PTM regulation as infected versus mock contrasts within each genotype at 12, 24, and 48 h. This time‐matched, within‐genotype design reduces, but does not completely eliminate, the influence of constitutive differences between genotypes. Indeed, a PCA of the total proteome indicates expected genotype‐specific baselines; however, direct genotype contrasts among time‐matched mock samples revealed comparatively modest baseline differences at the PTM level (phosphorylation and acetylation). Accordingly, our conclusions regarding pathogen‐induced regulation are based on changes relative to time‐matched mock controls within genotype.

Overall, our results provide a foundation for prioritizing targets that modulate immune‐related proteins, thereby refining the biological understanding of the rice–*M. oryzae* pathosystem. The targets proposed here should be subjected to functional validation to increase confidence in subsequent biotechnological strategies. Moreover, the hypothesis emerging from this study, that phosphorylation‐dominant regulation is linked to enhanced defense responses, whereas acetylation‐associated changes are linked to susceptibility‐related stress responses, needs to be tested across a larger set of genotypes contrasting for blast disease. Such testing will help validate diagnostic signatures for rapid assessment of genotype resistance levels and could accelerate breeding programs.

## MATERIALS AND METHODS

### Plant materials and experimental design

Rice (*Oryza sativa*) genotypes with contrasting resistance to *M. oryzae* infection were used: IRGA 409 (susceptible) and IRGA 424 (resistant). Cleaned seeds were germinated on paper rolls and maintained in a development growth chamber (Solab Cientifica, Piracicaba, Brazil) for 10 days. After that, the seedlings were transplanted into pots (two plants per pot) filled with commercial substrate and cultivated within a greenhouse under semi‐controlled environmental conditions (26–30°C, 80% relative humidity, and 700–850 μmol/m^2^/s of daylight).

The *M. oryzae* strain 787 was obtained from the Rio Grande do Sul Rice Institute (IRGA) and cultured on oatmeal agar (OAT) medium at 26°C under a 16‐h photoperiod for 15 days. Conidial suspensions were prepared as described by Molinari and Talbot (2022). Rice plants were uniformly sprayed with the conidial suspension (2 × 10^5^ conidia ml^−1^) containing 0.01% Tween 20 until runoff. Immediately after inoculation, the plants were enclosed in plastic bags to maintain high humidity and prevent the dispersion of fungal spores. The inoculation was carried out at the end of the day so that the plants were kept in the dark for 10 h (night). Infections were assessed at 12, 24, and 48 h post‐inoculation (hpi). Mock‐treated plants (sprayed with sterile water and 0.01% Tween 20) were sampled at the same time points as controls. Leaf samples were rapidly collected and immediately flash‐frozen in liquid nitrogen, then lyophilized. The time course selected for sample collection was based on previous studies. Meng et al. (2018) demonstrated that differentially regulated genes could be detected 6 h after *M. oryzae* infection, whereas Du et al. (2024) reported a similar activation approximately 8 h after infection, showing that early infection stages occur before the appearance of visible symptoms. These time points therefore represent early phases of host–pathogen interaction and allow the detection of pre‐symptomatic molecular changes.

The experiment consisted of three points, two treatment conditions (mock and infected), and two rice genotypes (susceptible and resistant). For each group, four independent biological replicates were collected, each consisting of pooled leaf tissue from two whole plants at the V5–V6 developmental stage. In total, the experimental design comprised 48 samples. The resistance level of these genotypes has been extensively characterized and is listed in the Technical Meeting Manual on Irrigated Rice Cultivation (Anon SOSBAI‐Sociedade Sul‐Brasileira de Arroz Irrigado, 2022).

### Determination of H_2_O_2_ and lipid peroxidation

The concentration of H_2_O_2_ was determined as proposed by Velikova et al. (2000). Leaf samples (200 mg) were ground and homogenized in 2.0 ml of 0.1% (w/v) trichloroacetic acid (TCA) and centrifuged at 12000 ***g*** for 15 min. To the supernatant (1.0 ml), 0.8 ml of 10 mM potassium phosphate buffer (pH 7.0) and 1.0 ml of 1 M potassium iodide were added. Samples' absorbances were read with a spectrophotometer at 390 nm, and the H_2_O_2_ concentration was calculated by comparison with the standard curve readings obtained from different H_2_O_2_ concentrations and expressed in μmol of H_2_O_2_.g^−1^ of fresh weight. Lipid peroxidation was determined by estimating the malondialdehyde content (MDA), according to the method by Heath and Packer (1968). Approximately 200 mg of fresh leaves were ground in 2.0 ml of trichloroacetic acid (TCA) (0.1% w/v) and centrifuged at 12000 ***g*** for 15 min. To the supernatant, a solution of thiobarbituric acid (TBA) (0.5% m/v) in TCA (10% w/v) was added and heated in a 95°C water bath for 30 min in closed tubes. Then, the reaction was paralyzed by flash‐cooling in an ice bath for 10 min. The absorbance of the resulting thiobarbituric acid‐reactive substances (TBA‐RS) was determined in a Multiskan SkyHigh Microplate Spectrophotometer (Thermo Scientific) at 535 nm and 600 nm. The concentration of the malondialdehyde (MDA)‐TBA complex was calculated by the equation: [MDA] = (A535–A600)/(ξb), where: ξ (extinction coefficient = 1.56 × 10^−5^ cm^−1^) and b: (optical length = 1 cm). Peroxidation was expressed in nmol of MDA g^−1^ of fresh weight.

### Protein extraction

Protein extraction and digestion into peptides were done using the Phenol‐FASP method (Song et al., 2018, 2020). Briefly, the total proteins were extracted from 15 mg of lyophilized rice leaves. Phenol Tris‐buffered pH 8 and an extraction buffer composed of Tris, EDTA, sucrose, and phosphatase inhibitors were used for extraction. The protein pellets were washed thrice using 0.1 M ammonium acetate in methanol, and an overnight precipitation was performed at −20C in 70% methanol. Extracted proteins were resuspended by sonication in urea resuspension buffer (8 M urea in 50 mM TRIS–HCl, pH = 7.0; 5 mM TCEP; 1× phosphatase inhibitor cocktail) and further cleaned through filter‐assisted sample preparation (FASP) using Amicon Ultra‐4 30 kDa MWCO filter units (Millipore) using a UA buffer (8 M urea in 100 mM TRIS–HCl, pH = 8.0; 1x phosphatase inhibitor cocktail). The FASP protocol involved multiple rounds of centrifugation and washing using UA buffer. Proteins were then reduced by 2 mM TCEP, alkylated using 50 mM iodoacetamide, and digested into peptides using one round of overnight incubation at 37°C with 1:100 (enzyme: protein) trypsin (Roche, Cat. No. 03708969001) and a second round of incubation for 4 h at 37°C with trypsin and Lys‐C (0.1 μg/μL) (Wako Chemicals, Catalog number 125‐05061). Purified samples were desalted using SepPack C18 columns (Waters) on a vacuum manifold (Montes et al., 2022).

### TMT labeling

Tandem Mass Tag labeling was performed using TMTpro 18plex reagents (ThermoFisher), as previously reported by Song et al. (2020); Montes et al. (2022). Each TMTpro 18‐plex label (175 μg) was resuspended in 65 μl anhydrous acetonitrile (ACN). For each sample, 130 μg peptide in 130 μl of 0.2 M HEPES (pH 8.5) was combined with 70 μl of its respective 18‐plex TMT label (final 35% ACN) and incubated for 2 h at room temperature. 1 μl of 5% hydroxylamine was added to a new tube, followed by 1 μl from each individual labeling reaction; the mixture was incubated for 15 min at room temperature. Then, 1.5 μl of the pooled test was diluted with 48.5 μl of 0.1% formic acid and analyzed by a single 2‐h LC–MS/MS run on an Orbitrap Exploris 480 to verify labeling efficiency. After confirming labeling efficiency >94%, individual samples were quenched with 5% hydroxylamine for 15 min. A second round of C18 desalting was performed after labeling. From each labeled sample, 100 μg was set aside for global proteome profiling; the remaining material was used for PTM enrichment.

Samples were multiplexed into three independent 18‐plex sets, each containing both genotypes (susceptible and resistant) and both treatments (mock and infected) for a single time point: Set 1: 12 h post‐infection (12 hpi); Set 2: 24 h post‐infection (24 hpi) and Set 3: 48 h post‐infection (48 hpi). All differential analyses were performed within each 18‐plex by comparing infected versus mock for the same genotype at the same time point. This within‐plex contrast mitigates batch effects and obviates the need for inter‐plex correction for the reported statistical tests. Because each plex contained the complete set of conditions required for the intended contrasts at that time point (both genotypes and both treatments), no channel‐bridging or inter‐plex normalization was required for our primary inferences.

### Post‐translation modifications (PTMs)—Phosphoproteome

Phosphopeptide enrichment was performed using both the High‐Select TiO2 Kit (Catalog number: A32993) and the High‐Select Fe‐NTA Kit (Catalog number: A32992) from Thermo Scientific. For each multiplex sample, peptides (2300 μg) were completely resuspended in 150 μl of Binding/Equilibration Buffer, followed by vortexing and verification of pH (<3). A centrifuge column adaptor was placed in a 2 ml collection tube, and the TiO2 spin tip was inserted into the adaptor. Next, 20 μl of wash buffer was added to the column and centrifuged at 3000 × ***g*** for 2 min. To equilibrate the column, 20 μl of Binding/Equilibration Buffer was applied and centrifuged under the same conditions. To bind the phosphopeptides, the 150 μl peptide suspension was applied to the spin tip and centrifuged at 1000 × ***g*** for 5 min. The sample was then reapplied to the column to improve phosphopeptide yield. The flow‐through was retained and dried for subsequent Fe‐NTA enrichment. For column washing, 20 μl of Binding/Equilibration Buffer was added and centrifuged at 3000 × ***g*** for 2 min, followed by another 20 μl of wash buffer under the same conditions. These two wash steps were repeated twice. For the final wash, 20 μl of LC–MS grade water was added, followed by centrifugation at 3000 × ***g*** for 2 min.

To elute the column, 50 μl of phosphopeptide elution buffer was added to the spin tip, followed by centrifugation at 1000 × ***g*** for 5 min. This step was repeated twice. The combined eluates were immediately dried in a speed vacuum concentrator to remove the elution buffer. The dried peptides were then resuspended in 20 μl of 0.1% formic acid for peptide quantification and subsequent LC–MS analysis. Following TiO2 enrichment, the High‐Select Fe‐NTA Phosphopeptide Enrichment Kit was applied to the retained lyophilized flow‐through samples. These peptides were resuspended in 200 μl of binding buffer, vortexed, and the pH was checked to ensure it was below 3. The samples were then added to the equilibrated spin column and gently mixed by tapping the bottom plug for 10 sec until the resin was fully suspended. The mixture was incubated for 30 min at room temperature, with gentle mixing every 10 min to maintain the resin in suspension. After incubation, the column was placed into a microcentrifuge tube and centrifuged at 1000 × g for 30 sec. The resulting flow‐through was dried and stored as backup. The column was then washed three times with 200 μl of wash buffer and once with LC–MS grade water. Elution was performed by adding 100 μl of Elution Buffer twice, and the combined eluates were immediately dried to remove the buffer. Finally, the dried peptides were resuspended in 40 μl of 0.1% formic acid. The enriched peptides obtained from both Fe‐NTA and TiO2 procedures were pooled for subsequent LC–MS analysis.

### Acetylome

Peptides were also enriched using anti‐acetylysine antibody‐conjugated agarose beads (PTM BIO, Cat PTM‐104) based on Walley et al. (2018). A separate set of peptides was prepared from the same tissue, TMT‐labeled and multiplexed, and then dried. Two milligrams per set were resuspended in 200 μl of IP buffer (100 mM NaCl, 1 mM EDTA, 20 mM Tris–HCl, pH 8.0). The insoluble peptides were pelleted by centrifugation, and the supernatant (pH 7–8) was carefully collected and kept on ice while the antibody preparation was carried out. Twenty microliters of antibody‐conjugated agarose beads were drained and washed three times with 500 μl of ice‐cold PBS. The peptide‐containing supernatant was then transferred to the tube containing the pre‐washed beads and incubated overnight at 4 °C with gentle end‐to‐end rotation. On the following day, the antibody–peptide complexes were pelleted by centrifugation at 500 ***g*** for 30 sec, and the supernatant was collected and stored. The antibody‐beads were washed three times with 500 μl of wash buffer (100 mM NaCl, 1 mM EDTA, 20 mM Tris–HCl, pH 8.0), each time inverting the tube gently for 15 sec, followed by centrifugation at 500 ***g*** for 30 sec. For the final wash, 250 μl of LC–MS grade water was added, and the beads were washed three times in the same manner. After the last wash, the beads were centrifuged at 500 g for 30 sec to pellet them.

The bound peptides were eluted three times using 100 μl of elution buffer (0.1% trifluoroacetic acid), each for 1 min with gentle end‐to‐end rotation at room temperature. The eluates were combined and centrifuged at 1000 × g for 1 min. The pooled eluate was then passed through a filter tube to remove any remaining beads. The enriched peptides were subsequently quantified, dried, and stored for downstream HPLC fractionation and mass spectrometry analysis.

### HPLC fractionation and LC–MS/MS

For global proteomics, a total of 50 μg of previously TMTpro‐labeled, pooled samples was subjected to offline high‐pH, reversed‐phase fractionation on an Ultimate 3000 UHPLC system (Thermo Scientific, Waltham, MA, USA). Twenty fractions were collected, and 1 μg from each fraction was used for LC–MS/MS analysis. For phosphoproteomics, a total of 12 μg of previously TMTpro‐labeled, pooled, and phospho‐enriched samples was subjected to offline high‐pH, reversed‐phase fractionation on an Ultimate 3000 UHPLC system (Thermo Scientific). Nine fractions were collected, and 1.2 μg from each fraction was used for LC–MS/MS analysis. For acetylproteomics, 2 μg of previously TMTpro‐labeled, pooled samples was used.

Chromatography was performed on a Thermo Vanquish Neo UHPLC in “heated trap‐and‐elute, backward flush” mode. Peptides were desalted and concentrated on a PepMap Neo trap column (300 μM i.d. × 5 mm, 5 μm C18, 100 Å μ‐Precolumn, Thermo Scientific) at a flow rate of 10 μl min^−1^. Sample separation was performed on a 110 cm Micro‐Pillar Array Column (μ‐PAC Neo, Thermo Scientific) with a flow rate of ~300 nl min^−1^ over a 120 min (global and phosphoproteome) or 180 min (acetylome) reverse phase active gradient. Followed by a column/trap wash at 80% ACN for 10 min. Eluted peptides were analyzed using a Thermo Scientific Orbitrap Exploris 480 mass spectrometer. For global proteome, a FAIMS pro Duo interface directly coupled to the UHPLC through an Easy Spray Ion source (Thermo Scientific) was installed. Data dependent acquisition was obtained using Xcalibur 4.0 software in positive ion mode with a spray voltage of 2.1 kV, a capillary temperature of 280°C, and RF of 45. MS1 spectra were measured at a resolution of 120 000 for global, and 60 000 for phosphoproteome/acetylome. An MS1 automatic gain control (AGC) of 3e6 with auto maximum ion time, and a mass range of 400–1400 m/z (global), 350–1400 m/z (phosphor/acetyl).

For global proteome, a cycle time of 0.8 sec was used to capture triggered MS2 at a resolution of 15 000 with the “Turbo TMTpro” setting on. A fixed first mass of 110 m/z. An AGC of 1e5 with a maximum ion time of 22 ms, a normalized collision energy of 33, and an isolation window of 0.7 m/z were used. Charge inclusion was set to 2–6. MS1 that triggered MS2 scans were dynamically excluded for 30 sec. FAIMS compensation voltages of −45 and −60, and a total carrier gas flow of 4.6 L/min was used. For Phospho and Acetyl proteomes, up to 30 MS2 were triggered at a resolution of 45 000. A fixed first mass of 100 m/z. An AGC of 3e5 with a maximum ion time of 96 ms, a normalized collision energy of 33, and an isolation window of 0.7 m/z were used. Charge inclusion was set to 2–6. MS1 that triggered MS2 scans were dynamically excluded for 20 sec.

Eluted peptides were analyzed using a Thermo Scientific Orbitrap Exploris 480 mass spectrometer. Data dependent acquisition was obtained using Xcalibur 4.0 software in positive ion mode with a spray voltage of 2.2 kV and a capillary temperature of 275°C and an RF of 60. MS1 spectra were measured at a resolution of 70 000, with an automatic gain control (AGC) of 3 × 10^6^, a maximum ion time of 100 ms, and a mass range of 400–2000 m/z. Up to 15 MS2 were triggered at a resolution of 17 500 or 35 000 was used for two replicate runs respectively. Note that the TMTpro labels used here have 1 Da spacing between reporter ion, which enables acquisition with the 17 500 resolution setting. A fixed first mass of 120 m/z was used. An AGC of 1e5 with a maximum ion time of 50 ms, an isolation window of 1.3 m/z, and a normalized collision energy of 31 were used. Charge exclusion was set to unassigned, 1, 5–8, and >8. MS1 scans that triggered MS2 scans were dynamically excluded for 25 sec.

### Proteomics data analysis

Spectra were searched using the Andromeda search engine (Cox et al., 2011) integrated into MaxQuant software (version 1.6.1.0; Tyanova et al., 2016). The search was performed against the *Oryza sativa* protein database (IRGSP‐1.0_protein_2024‐01‐11), which was automatically supplemented by MaxQuant with reverse decoy sequences and a list of common contaminants. Carbamidomethylation of cysteine residues was specified as a fixed modification, while methionine oxidation and N‐terminal acetylation of proteins were set as variable modifications. In experiments targeting PTMs, lysine acetylation (K) and phosphorylation on serine, threonine, and tyrosine residues (STY) were also included as variable modifications. Digestion parameters were set to “specific” with the protease combination Trypsin/P:LysC, allowing up to two missed cleavages. For TMT‐labeled samples, the sample type was set to “Reporter Ion MS2,” and the TMTpro18plex option was selected for both lysine residues and peptide N‐termini. A false discovery rate (FDR) of less than 1% was applied at both the peptide‐spectrum match (PSM) and protein identification levels. The “second peptide” option was enabled to identify co‐fragmented peptides. The “match between runs” feature was not used in the analysis.

Statistical analysis for global proteomics was performed using TMT‐NEAT Analysis Pipeline version 1.4 (Clark et al., 2021). Differential protein accumulation analysis (DAP) between control and inoculated plants was performed using the Pseq algorithm from the PoissonSeq package (Li et al., 2012). A FDR cutoff of q‐value <0.1 was used for designating DAPs. For phosphoproteome and acetylome analyses, statistical processing was performed using Perseus (version 2.1.0.0). Prior to statistical testing, the following data preprocessing steps were applied: (i) only modification sites with a localization probability greater than 0.75 were retained; (ii) missing values were imputed based on a normal distribution to simulate low‐abundance signals; and (iii) data were log2‐transformed to stabilize variance. Differential expression analysis was performed in Perseus and R using the Limma and DEqMS packages (Zhu et al., 2020), with a q‐value threshold of <0.1 and log_2_(fold change) >0.5.

### Biological downstream analysis of differentially accumulated proteins and PTMs from the global proteome, phosphoproteome, and acetylome

Gene Ontology (GO) terms are widely used as a complementary approach for gene function annotation and have been extensively applied to gain deeper insights into the biological processes underlying specific adverse conditions in phenotype‐driven experiments, such as phosphoproteomics and acetylomics (Guo, Chai, et al., 2022). In this study, because the RAP‐DB genome is partially (45–50%) annotated with GO terms, we annotated this feature using GOMAP (Wimalanathan & Lawrence‐Dill, 2021), achieving 100% coverage. GO annotation was performed using a single representative protein for each gene, the longest one. To identify the main GO terms associated with the three GO categories, biological processes, molecular functions, and cellular components, we performed enrichment analysis using Fisher's exact test implemented in a custom R script. Enrichment analysis was conducted separately for up‐ and downregulated protein datasets. GO terms with an FDR <0.05 were considered significantly enriched. Enriched GO terms were visualized using bubble charts generated with ggplot2.

For motif analysis, amino acid sequence windows were extracted from differentially regulated phosphoproteins and acetylated proteins at 12, 24, and 48 h post‐inoculation for each genotype. Eleven‐residue fragments were retrieved, consisting of five amino acids upstream, the central modified residue (S/T/Y for phosphorylation or K for acetylation), and five residues downstream. Frequency distributions of S/T/Y‐centered and K‐centered motifs were calculated, and the relative abundance (%) of each residue type at each position was determined. All analyses were conducted in R version 4.3.3 using the ggseqlogo package (Wagih, 2017).

For phospho‐conservation analyses, we used a set of DAPs that were differentially phosphoregulated in this study, and their orthologs in *Z. mays*, *A. thaliana*, and *S. bicolor* were identified using the OMA (Orthologous Matrix) software. Orthologous groups containing the phosphorylated proteins of interest were used for phylogenetic inference, followed by manual curation to define the final ortholog sets. Proteins lacking representatives in these species were removed. The phosphorylation sites of interest were extracted from the additional species using MAFFT alignments, and the site conservation percentage was subsequently calculated.

### Kinase–substrate prediction analysis

To infer kinase–substrate relationships from the phosphoproteomic dataset, we implemented a motif‐based prediction strategy using custom scripts in R using Biostrings, BiocManager and Tidyverse packages. Phosphosites that were differentially regulated across genotypes and time points were selected based on fold change (1.4 fold), adjusted *P*‐value (<0.1), and a site localization probability greater than 0.75. Only sites showing statistically relevant changes in phosphorylation dynamics were retained for downstream motif‐based kinase prediction. Each selected phosphosite was mapped to its corresponding sequence window (±7 amino acids surrounding the modified residue), and only complete 15‐mer sequences were considered. A curated set of kinase recognition motifs (available in Data S16 and S17) was manually compiled from the literature, representing consensus phosphorylation patterns associated with key plant kinase families, including MAPKs (Mitogen‐activated protein kinase), CDPKs (Calcium‐dependent protein kinase), CK2 (Casein kinase 2), SnRK2s (SNF1‐related protein kinase 2), RLKs (Receptor‐like kinase), AGC (AGC protein kinases), and CAMKs (Ca^2+^/calmodulin‐dependent protein kinase). These motifs were encoded as regular expressions derived from experimentally characterized substrate preferences reported in previous studies (e.g., Hamel et al., 2014; Meggio & Pinna, 2003; Vlad et al., 2008; Yoon & Seger, 2006; Zhang et al., 2002). No external motif prediction database was used. Instead, predictions were restricted to differentially phosphorylated sites whose surrounding sequences matched these canonical recognition motifs. Motif scanning was performed by systematically comparing each 15‐mer phosphopeptide against the full set of regular expressions. Kinase–substrate associations were assigned when a consensus motif was matched, allowing the construction of directed kinase–substrate networks for downstream temporal and genotype‐specific analyses. The predicted interactions were used to construct a directed kinase–substrate network using Cytoscape version 3.10.3 (Shannon et al., 2003), and the Network Analyzer package was used to compute the degree centrality (i.e., the Network Analyzer package was used to calculate the degree centrality) (i.e., the number of edges) for each node. To add contextual validation and reduce the speculative nature of the motif‐based predictions, an additional co‐localization analysis was performed by examining the subcellular localization of each kinase in UniProt/GO: Cellular Component, as well as the localization of the putative substrates discussed in the manuscript, assessing whether they were compatible. The kinases classified as “CK2,” retrieved using the search term “casein kinase ii *Oryza sativa*” in UniProt, were found to localize predominantly to the cytosol and nucleus. The kinases grouped under “CAMK,” identified using the term “calmodulin binding *Oryza sativa* kinase,” were mainly localized to the cytosol, nucleus, and plasma membrane. The “SnRK2” kinases, retrieved with the term “SnRK2 *Oryza sativa*,” were predominantly localized to the cytosol and nucleus.

## AUTHOR CONTRIBUTIONS

PAA, MCS‐F, and JW conceived and designed the experiments. PAA performed the experiments. PAA, GS, and CM‐S prepared samples for proteomics. CM‐S conducted the MS runs and performed the MaxQuant analysis. PAA and LWPA carried out data analysis and bioinformatics. MCS‐F and JW provided reagents, materials, and analysis tools. PAA wrote the manuscript. All authors reviewed and approved the final version of the manuscript.

## CONFLICT OF INTEREST

The authors declare that they have no conflicts of interest.

## Supporting information


**Figure S1.** Visual symptoms observed in IRGA 409 (susceptible) and IRGA 424 (resistant) genotypes at 3 and 14 days after inoculation with *Magnaporthe oryzae*. *M. oryzae* spores and culture are also shown.

**Figure S2.** Phosphoproteome Quantification and Phosphorylation Site Composition.

**Figure S3.** Conserved Phosphorylation Motifs in IRGA 409.

**Figure S4.** Conserved Phosphorylation Motifs in IRGA 424.

**Figure S5.** Acetylome Quantification and Acetylation Types.

**Figure S6.** Conserved Acetylated Motifs in IRGA 409 and IRGA 424.

**Figure S7.** Phosphosite conservation analysis. (A) Phylogenetic analysis of differentially accumulated proteins (DAP) proteins from.

**Data S1.** Global results—Total and Differentially Accumulated Proteins.

**Data S2.** Phosphoproteome results—Phosphoproteome profiles, Number of phosphosites, Total and Differentially Regulated Phosphosites of rice genotypes (IRGA 409 and IRGA 424) at 12, 24, and 48 h after inoculation with *Magnaporthe oryzae*.

**Data S3.** Acetylome results—Acetylome profiles, Total and Differentially Regulated Acetylsites of rice genotypes (IRGA 409 and IRGA 424) at 12, 24, and 48 h after inoculation with *Magnaporthe oryzae*.

**Data S4‐S15.** The complete tables of acetylated proteins in IRGA 409 and IRGA 424 at 12, 24 and 48 hpi.

**Data S16.** Kinase Motifs.

**Data S17.** Kinase Prediction.

**Data S18.** Differentially accumulated proteins (DAP) and post‐translational modifications (PTM) regulations.

## Data Availability

The original MS proteomics raw data, as well as the MaxQuant output files, may be downloaded from MassIVE (http://massive.ucsd.edu) using the identifier: MSV000099743.
