# Supplementary figures and images for: Multi‐proteomic profiling indicates potential regulatory signatures underlying rice resistance to Magnaporthe oryzae
Source: Plant J. 2026 Apr 21;126(2):e70892. doi: 10.1111/tpj.70892 (PMC13099112; doi:10.1111/tpj.70892)

3DPI\_409

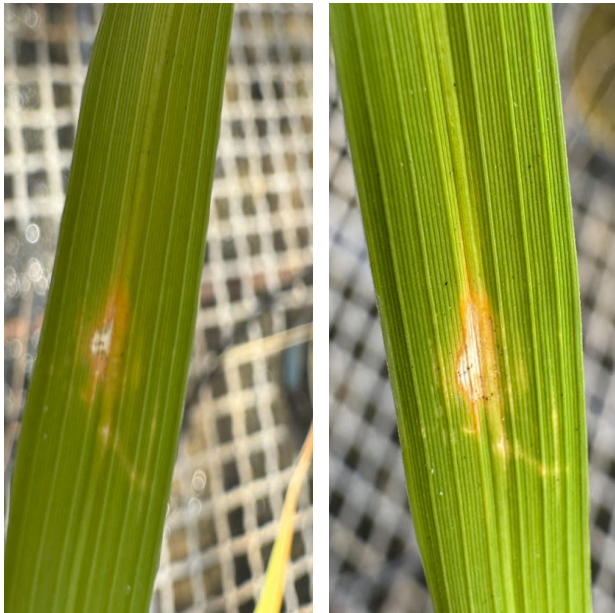

14DPI\_409

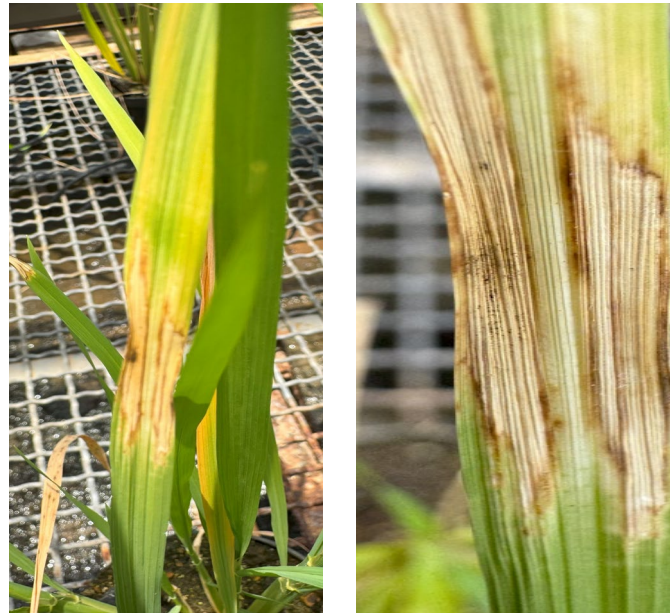

M. O sporos

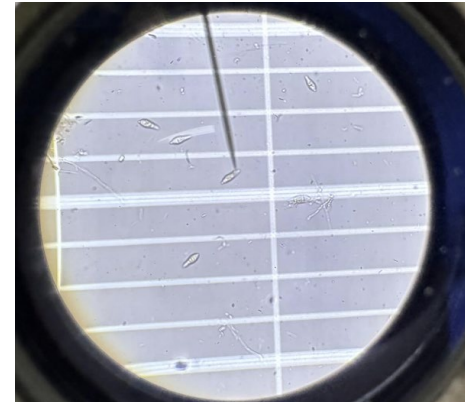

M. O culture

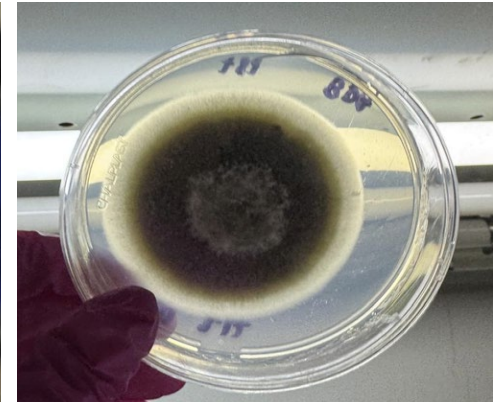

3DPI\_424

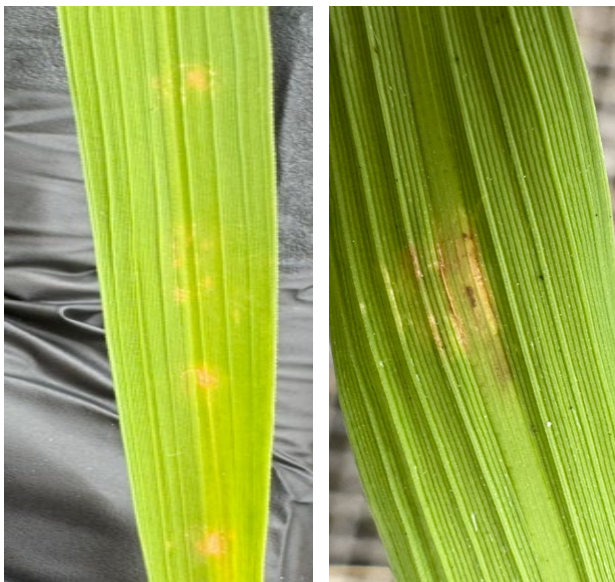

14DPI\_424

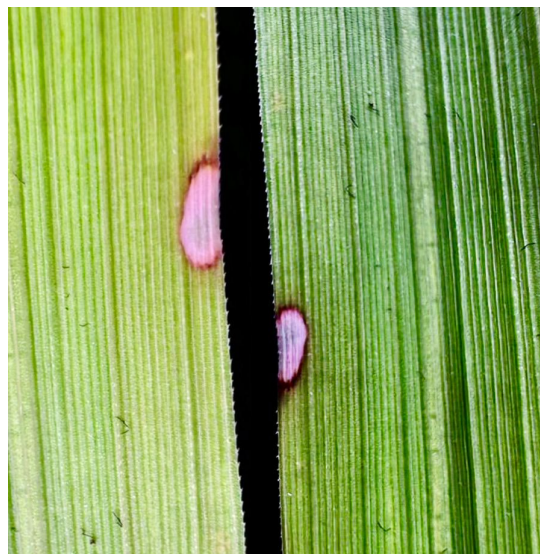

409

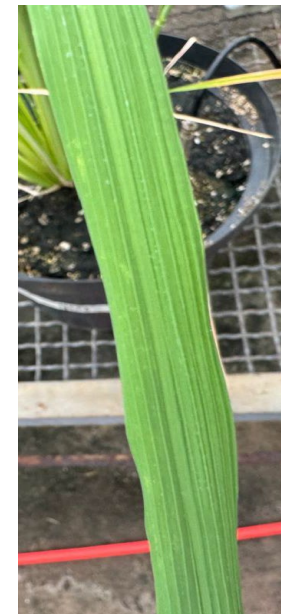

424

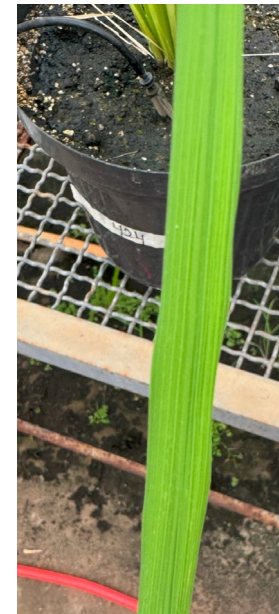

Control

Supplement: Supplementary file 1 — Figure S1. Visual symptoms observed in IRGA 409 (susceptible) and IRGA 424 (resistant) genotypes at 3 and 14 days after inoculation with Magnaporthe oryzae. M. oryzae spores and culture are also shown. [file TPJ-126-0-s014.pdf]

a)

### Phosphoproteome Quantification

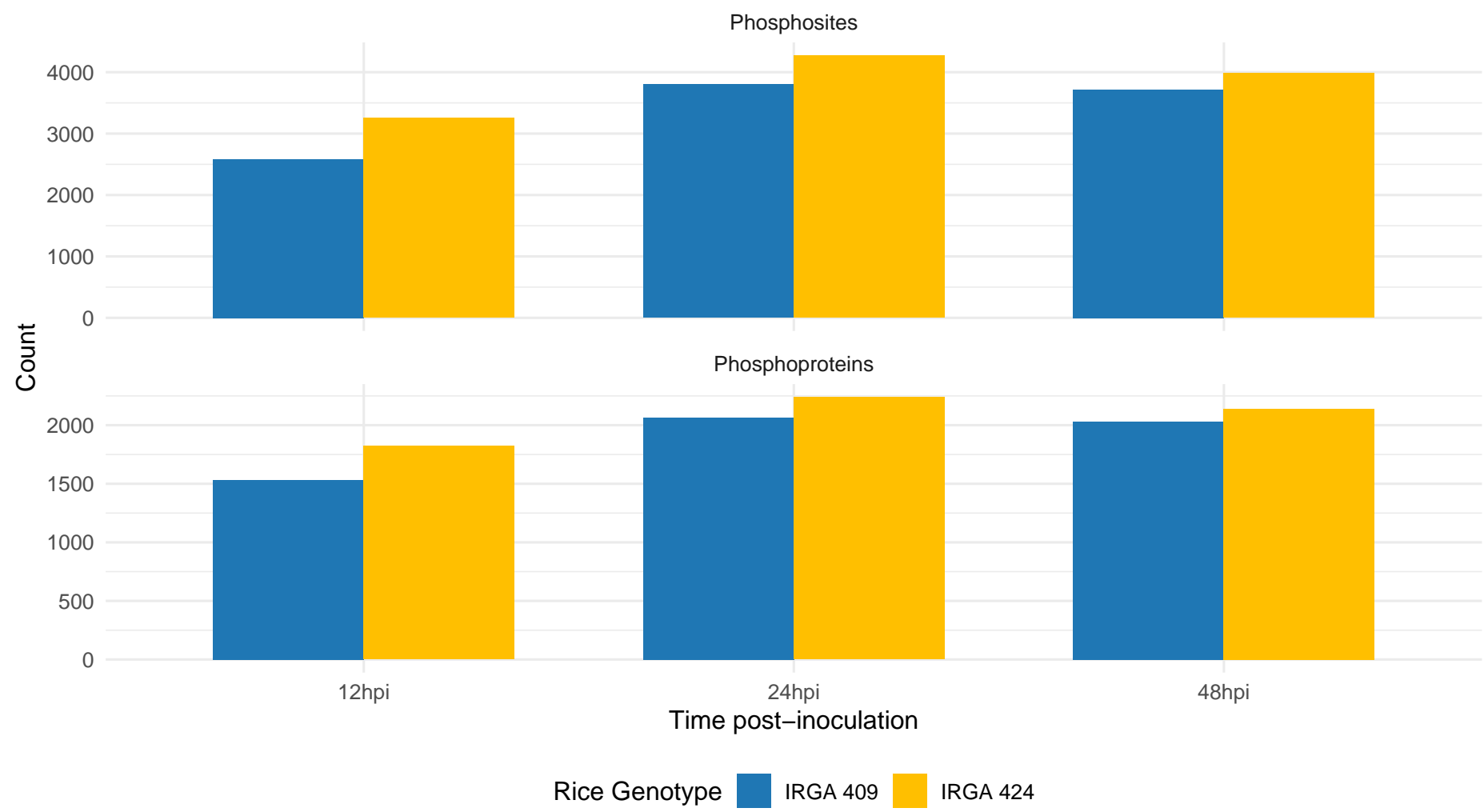

b)

### Phosphorylation Site Composition

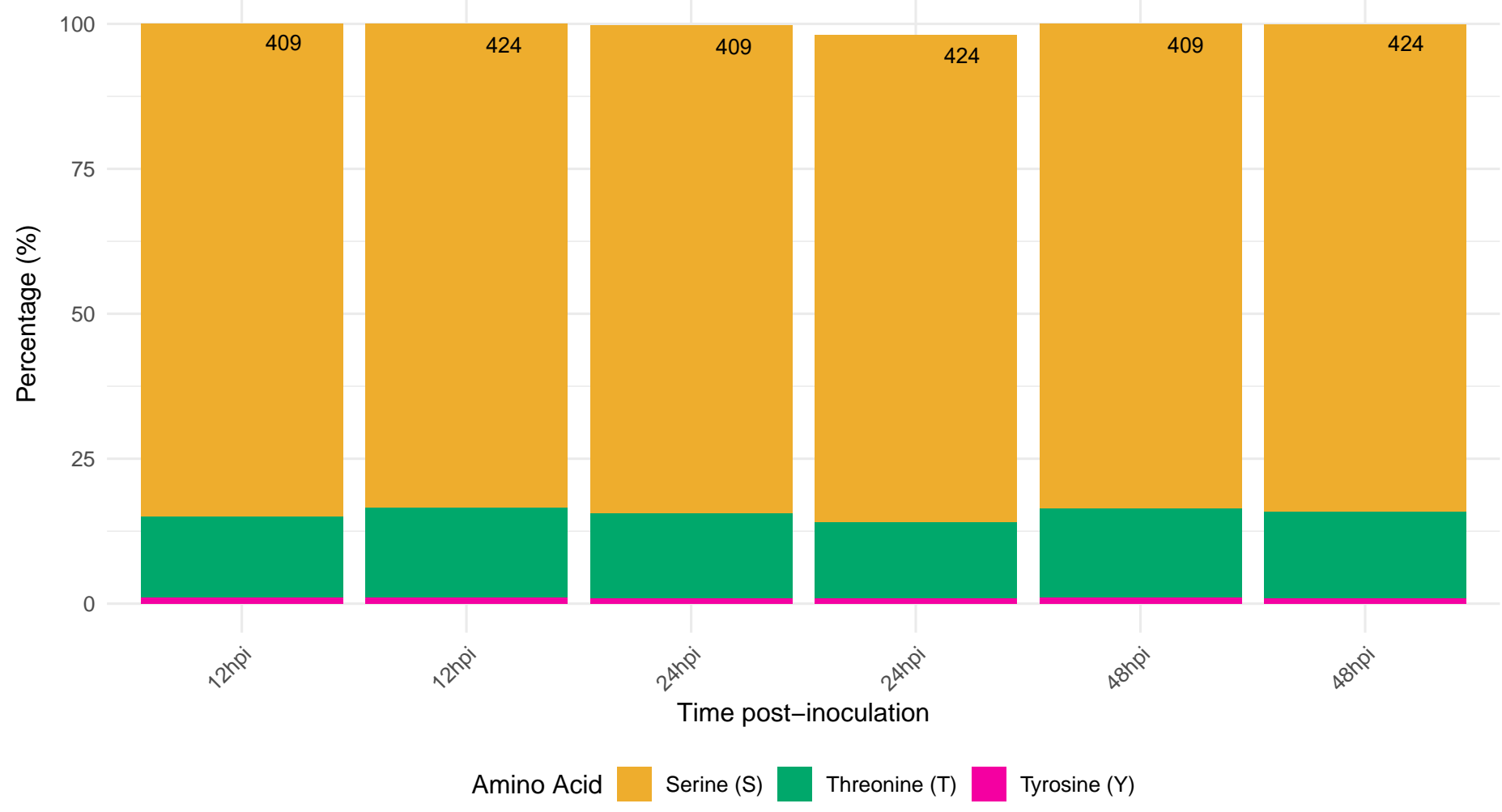

Supplement: Supplementary file 2 — Figure S2. Phosphoproteome Quantification and Phosphorylation Site Composition. [file TPJ-126-0-s001.pdf]
