# Supplementary material for: Multi‐proteomic profiling indicates potential regulatory signatures underlying rice resistance to Magnaporthe oryzae
Source: Plant J. 2026 Apr 21;126(2):e70892. doi: 10.1111/tpj.70892 (PMC13099112; doi:10.1111/tpj.70892)
Supplement: Supplementary file 3 — Figure S3. Conserved Phosphorylation Motifs in IRGA 409. [file TPJ-126-0-s007.pdf]

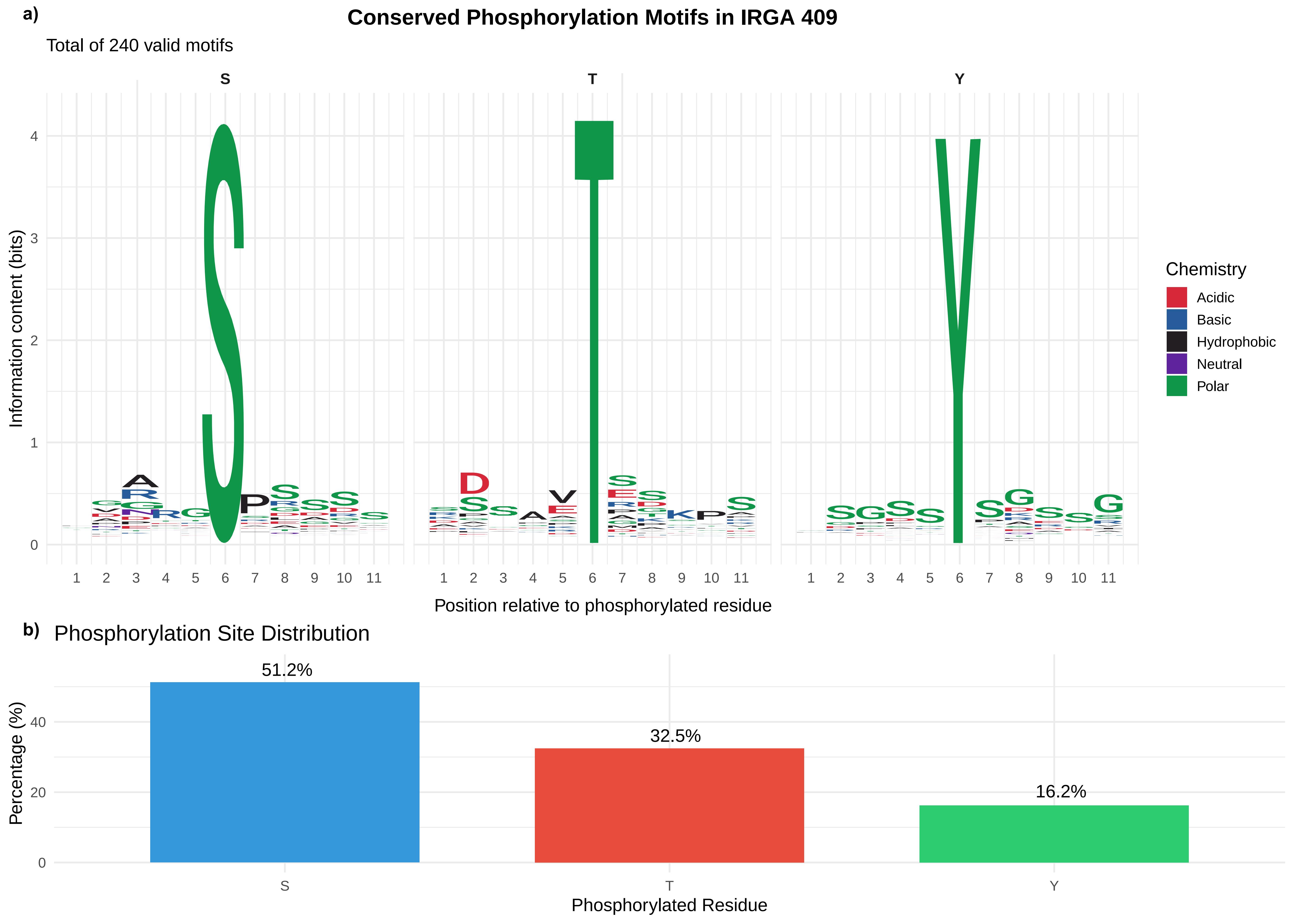
a)
Conserved Phosphorylation Motifs in IRGA 409
Total of 240 valid motifs
S
T
Y
Information content (bits)
0
1
2
3
4
1 2 3 4 5 6 7 8 9 10 11
Position relative to phosphorylated residue
Chemistry
Acidic
Basic
Hydrophobic
Neutral
Polar
b)
Phosphorylation Site Distribution
51.2%
32.5%
16.2%
Percentage (%)
0
20
40
S
T
Y
Phosphorylated Residue
