# Supplementary material for: Multi‐proteomic profiling indicates potential regulatory signatures underlying rice resistance to Magnaporthe oryzae
Source: Plant J. 2026 Apr 21;126(2):e70892. doi: 10.1111/tpj.70892 (PMC13099112; doi:10.1111/tpj.70892)
Supplement: Supplementary file 4 — Figure S4. Conserved Phosphorylation Motifs in IRGA 424. [file TPJ-126-0-s004.pdf]

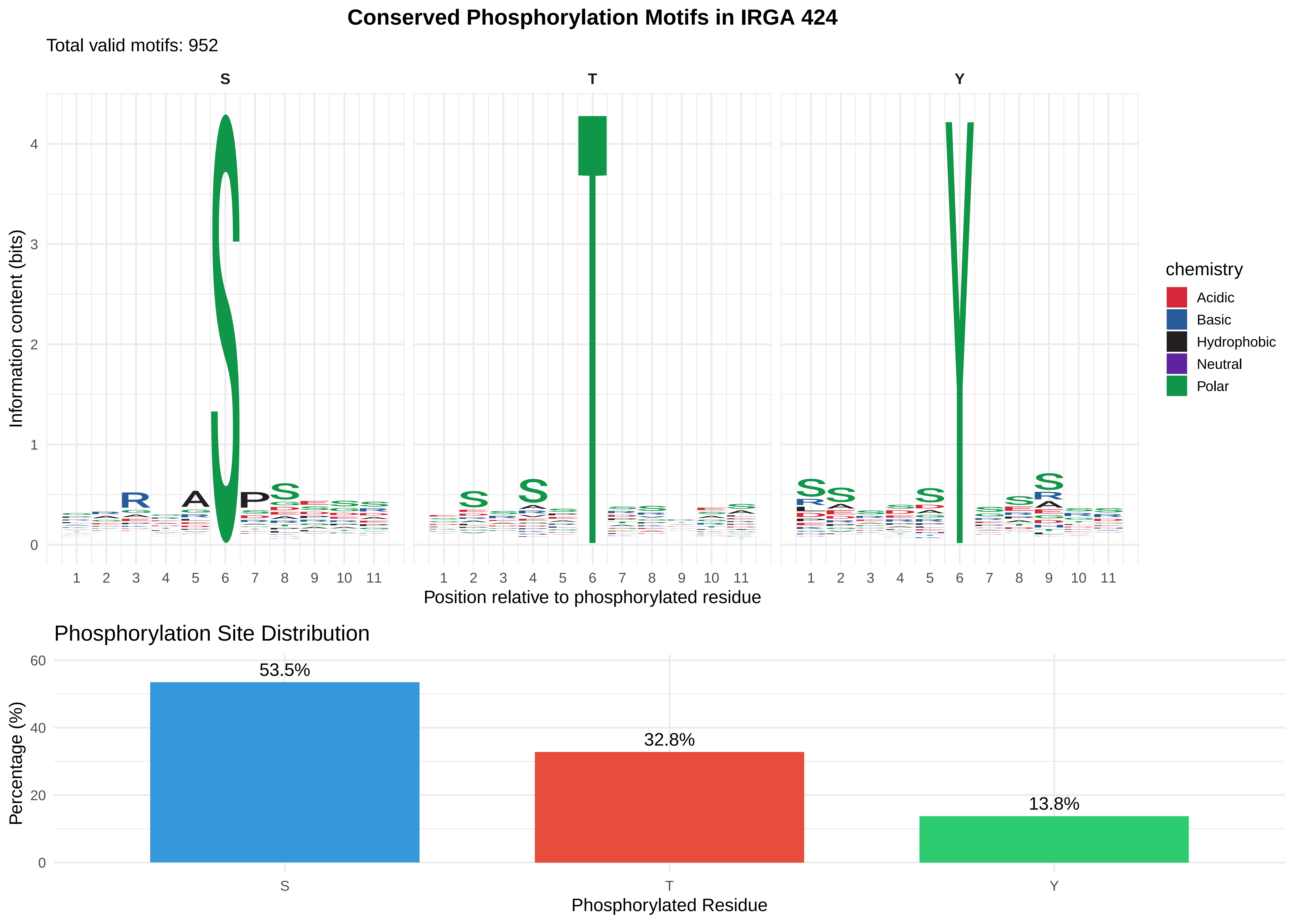

Conserved Phosphorylation Motifs in IRGA 424
Total valid motifs: 952
S
T
Y
Information content (bits)
0
1
2
3
4
1 2 3 4 5 6 7 8 9 10 11
Position relative to phosphorylated residue
chemistry
Acidic
Basic
Hydrophobic
Neutral
Polar
Phosphorylation Site Distribution
Percentage (%)
0
20
40
60
53.5%
32.8%
13.8%
S
T
Y
Phosphorylated Residue
