# Supplementary material for: Multi‐proteomic profiling indicates potential regulatory signatures underlying rice resistance to Magnaporthe oryzae
Source: Plant J. 2026 Apr 21;126(2):e70892. doi: 10.1111/tpj.70892 (PMC13099112; doi:10.1111/tpj.70892)
Supplement: Supplementary file 5 — Figure S5. Acetylome Quantification and Acetylation Types. [file TPJ-126-0-s008.pdf]

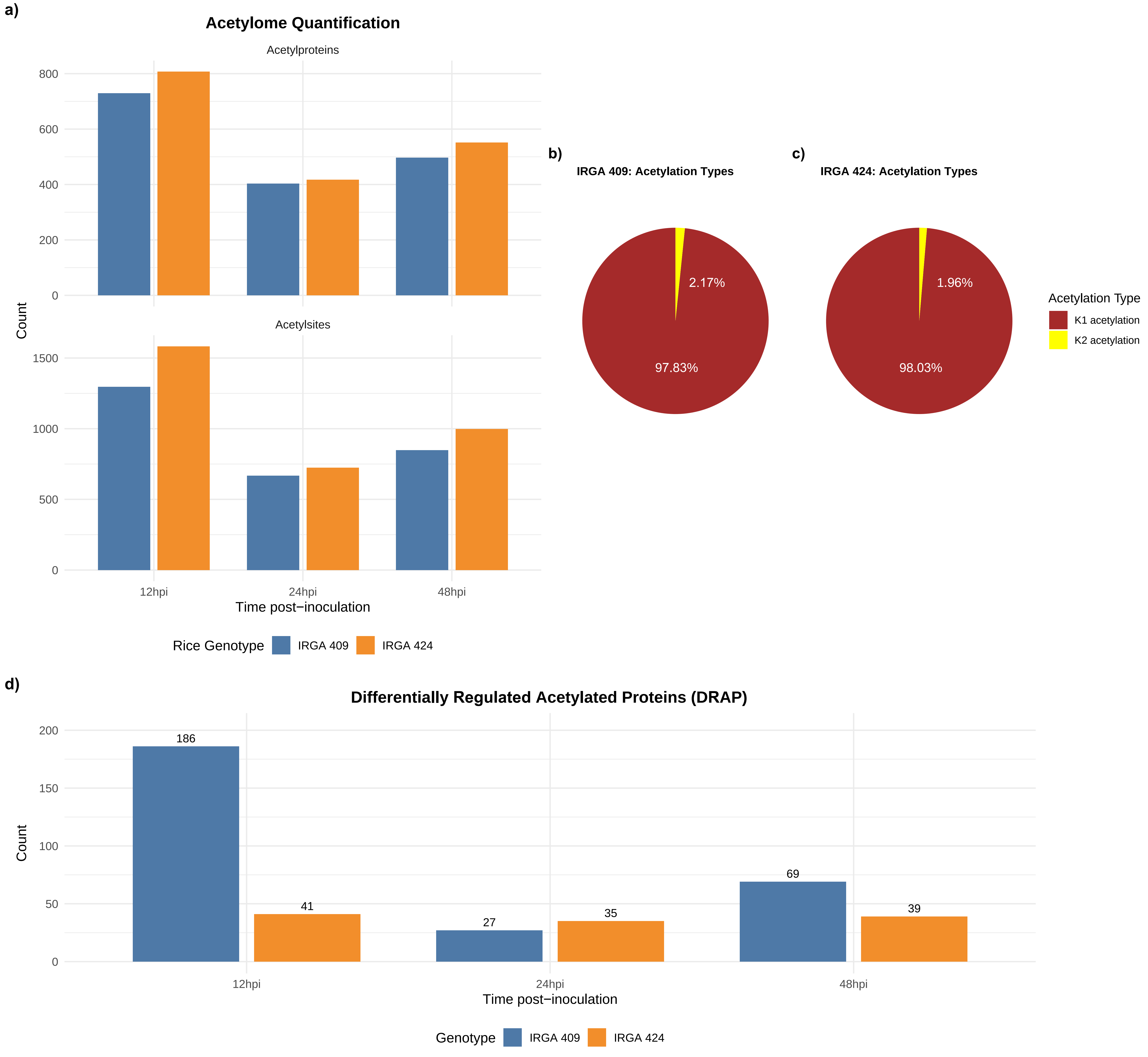
a)
Acetylome Quantification
Acetylproteins
Acetylsites
Count
800
600
400
200
0
1500
1000
500
0
12hpi
24hpi
48hpi
Time post−inoculation
Rice Genotype
IRGA 409
IRGA 424
b)
IRGA 409: Acetylation Types
2.17%
97.83%
c)
IRGA 424: Acetylation Types
1.96%
98.03%
Acetylation Type
K1 acetylation
K2 acetylation
d)
Differentially Regulated Acetylated Proteins (DRAP)
Count
200
150
100
50
0
186
41
27
35
69
39
12hpi
24hpi
48hpi
Time post−inoculation
Genotype
IRGA 409
IRGA 424
