# Supplementary material for: Multi‐proteomic profiling indicates potential regulatory signatures underlying rice resistance to Magnaporthe oryzae
Source: Plant J. 2026 Apr 21;126(2):e70892. doi: 10.1111/tpj.70892 (PMC13099112; doi:10.1111/tpj.70892)
Supplement: Supplementary file 6 — Figure S6. Conserved Acetylated Motifs in IRGA 409 and IRGA 424. [file TPJ-126-0-s012.pdf]

a)

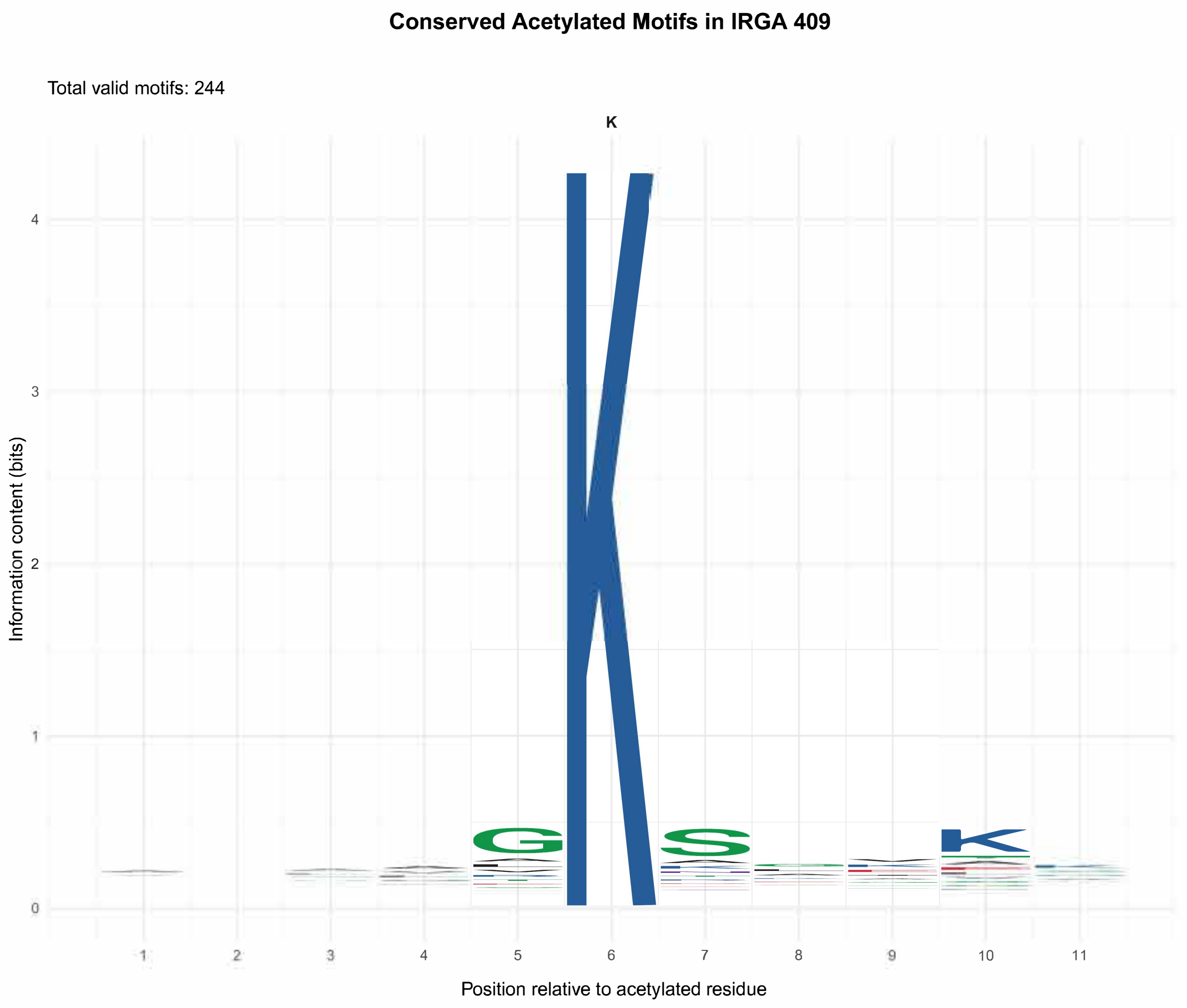
Conserved Acetylated Motifs in IRGA 409
Total valid motifs: 244
K
Information content (bits)
0
1
2
3
4
1
2
3
4
5
6
7
8
9
10
11
Position relative to acetylated residue

b)

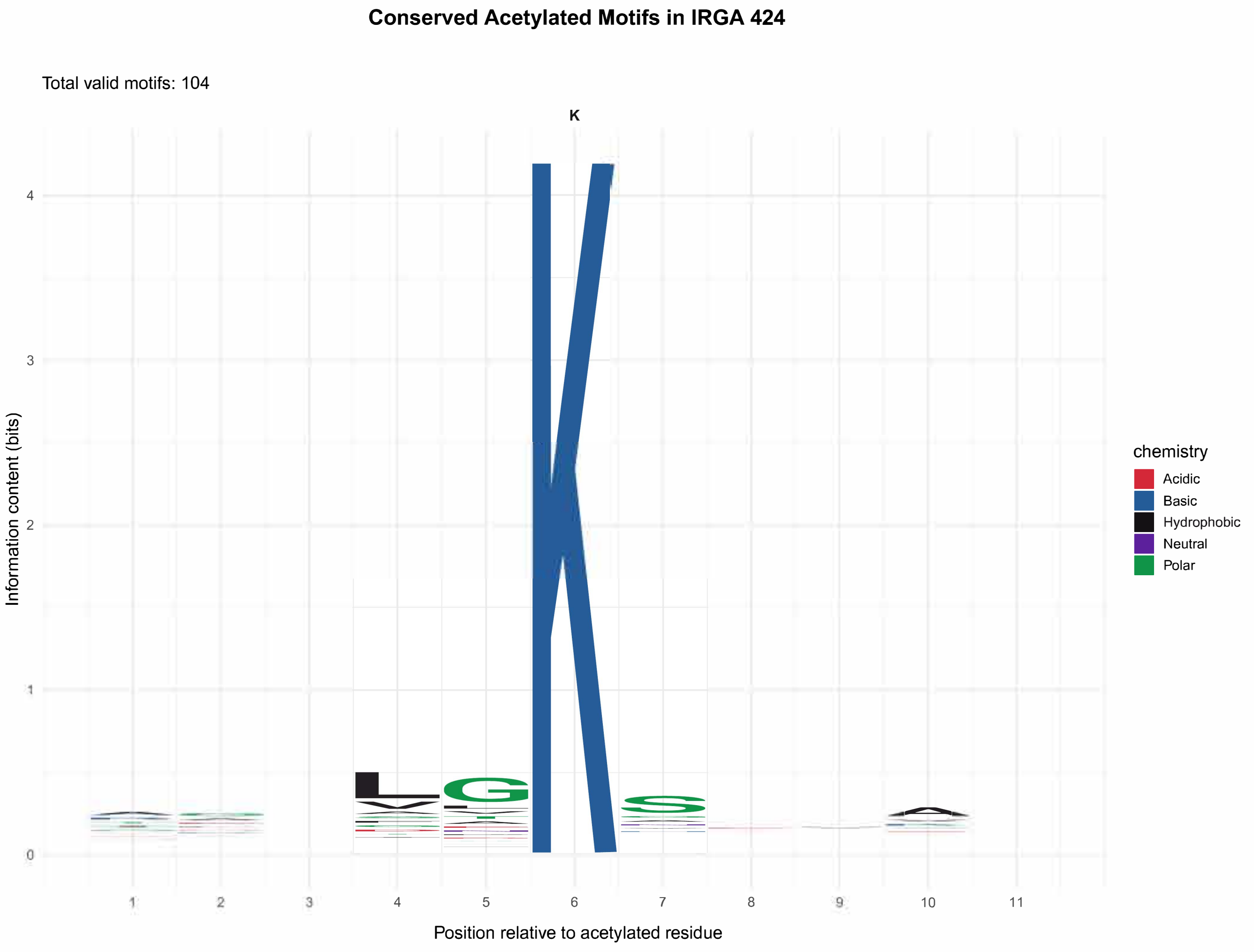
Conserved Acetylated Motifs in IRGA 424
Total valid motifs: 104
K
Information content (bits)
0
1
2
3
4
1
2
3
4
5
6
7
8
9
10
11
Position relative to acetylated residue
chemistry
Acidic
Basic
Hydrophobic
Neutral
Polar
