# Supplementary material for: Multi‐proteomic profiling indicates potential regulatory signatures underlying rice resistance to Magnaporthe oryzae
Source: Plant J. 2026 Apr 21;126(2):e70892. doi: 10.1111/tpj.70892 (PMC13099112; doi:10.1111/tpj.70892)
Supplement: Supplementary file 7 — Figure S7. Phosphosite conservation analysis. (A) Phylogenetic analysis of differentially accumulated proteins (DAP) proteins from. [file TPJ-126-0-s003.pdf]

A)

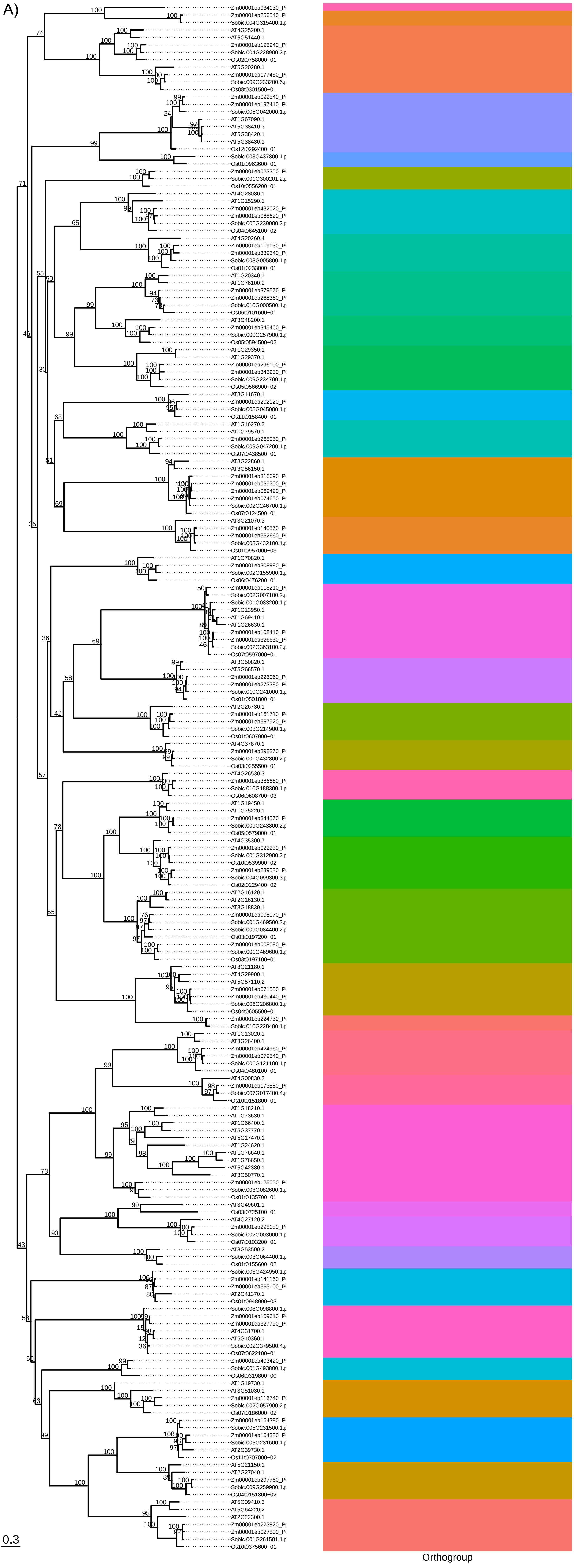

B)

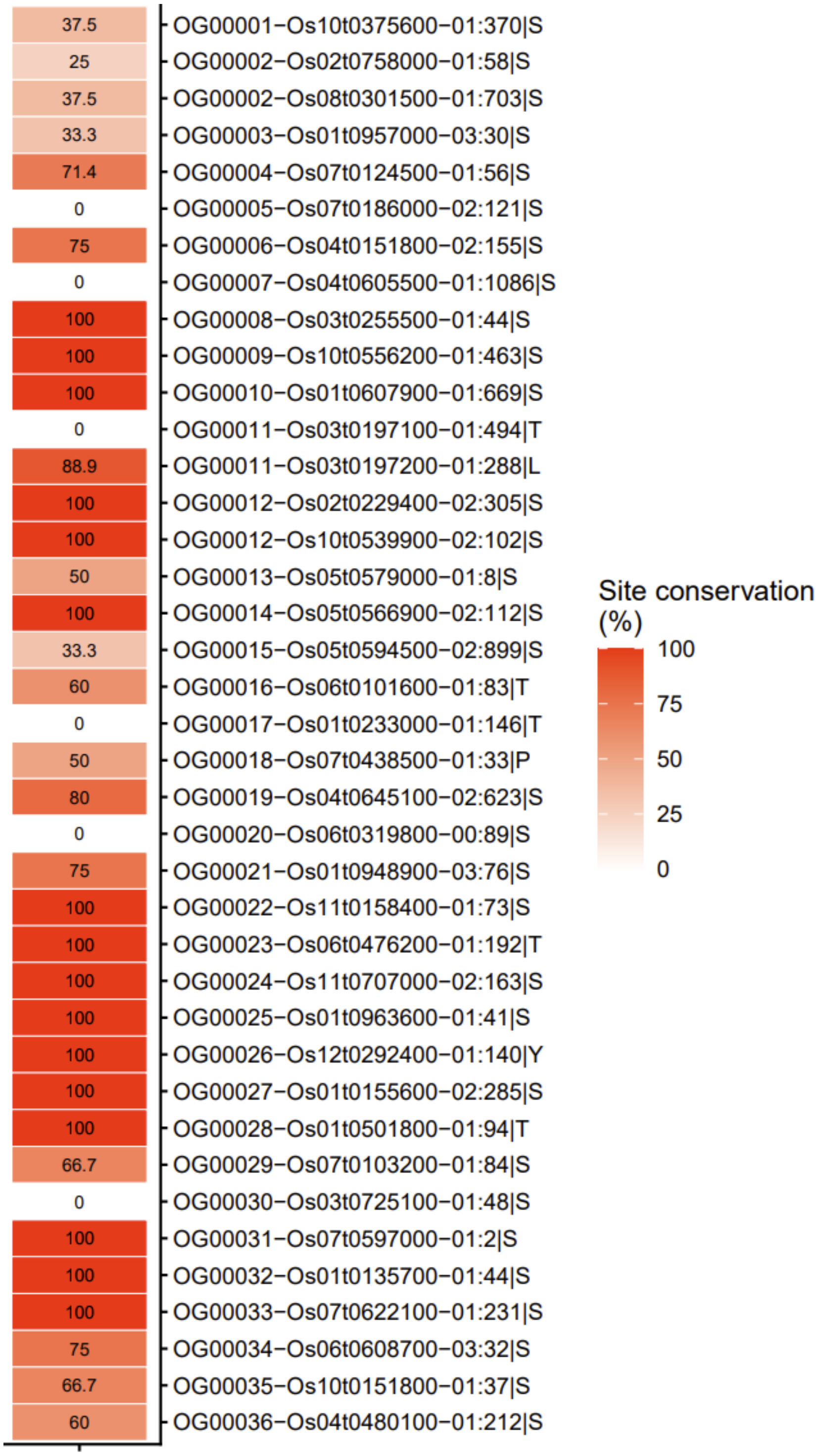

Figure S7. Phosphosite-conservation analysis. (A) Phylogenetic analysis of DAP proteins from *Oryza sativa* with orthologs in *Zea mays*, *Arabidopsis thaliana*, and *Sorghum bicolor*. (B) Phosphosite-conservation percentage across species orthologs.
